# The Hepatitis C Virus-Induced Membranous Web and Associated Nuclear Transport Machinery Limit Access of Pattern Recognition Receptors to Viral Replication Sites

**DOI:** 10.1371/journal.ppat.1005428

**Published:** 2016-02-10

**Authors:** Christopher J. Neufeldt, Michael A. Joyce, Nicholas Van Buuren, Aviad Levin, Karla Kirkegaard, Michael Gale Jr., D. Lorne J. Tyrrell, Richard W. Wozniak

**Affiliations:** 1 Department of Cell Biology University of Alberta, Edmonton, Alberta, Canada; 2 Department of Medical Microbiology and Immunology, University of Alberta, Edmonton, Alberta, Canada; 3 Li Ka Shing Institute of Virology, Edmonton, Alberta, Canada; 4 Department of Genetics, Stanford University School of Medicine, Stanford, California, United States of America; 5 Department of Immunology, University of Washington, Seattle, Washington, United States of America; Purdue University, UNITED STATES

## Abstract

Hepatitis C virus (HCV) is a positive-strand RNA virus of the *Flaviviridae* family and a major cause of liver disease worldwide. HCV replicates in the cytoplasm, and the synthesis of viral proteins induces extensive rearrangements of host cell membranes producing structures, collectively termed the membranous web (MW). The MW contains the sites of viral replication and assembly, and we have identified distinct membrane fractions derived from HCV-infected cells that contain replication and assembly complexes enriched for viral RNA and infectious virus, respectively. The complex membrane structure of the MW is thought to protect the viral genome limiting its interactions with cytoplasmic pattern recognition receptors (PRRs) and thereby preventing activation of cellular innate immune responses. Here we show that PRRs, including RIG-I and MDA5, and ribosomes are excluded from viral replication and assembly centers within the MW. Furthermore, we present evidence that components of the nuclear transport machinery regulate access of proteins to MW compartments. We show that the restricted assess of RIG-I to the MW can be overcome by the addition of a nuclear localization signal sequence, and that expression of a NLS-RIG-I construct leads to increased immune activation and the inhibition of viral replication.

## Author Summary

Hepatitis C virus (HCV) is a positive-strand RNA virus and it is a major cause of liver disease worldwide affecting more than 170 million individuals. Infection of cells with HCV leads to rearrangement of cytoplasmic host cell membranes and the formation of the membranous web (MW) containing viral replication and assembly complexes. The MW is thought to function in concentrating viral components, regulating virus replication, and immune evasion. Our analysis has provided new insight into the organization of the MW and the mechanisms that contribute to the formation and maintenance of distinct compartments within the MW. We show that the MW limits access of host cell innate immune receptors to sites of viral replication and assembly. Moreover, we show that components of the nuclear transport machinery, normally involved in regulating traffic between the cytoplasm and the nucleus, have a role in limiting immune receptor access to compartments within the MW. These findings provide important insights in how HCV, and likely other positive-strand RNA viruses, organize their replication factories and evaded recognition by host cell immune receptors.

## Introduction

Positive-strand RNA viruses account for a significant portion of the total viral diseases affecting humans around the world. Within this class of viruses is the *Flaviviridae* family, consisting of four viral genera, including *Flavivirus* and *Hepacivirus*. HCV is a *Hepacivirus* that is estimated to infect 170 million people world-wide, and, without treatment, this virus leads to end stage liver disease in approximately 30% of patients [1]. The replication cycle of HCV occurs primarily in the cytoplasm of host cells where, upon entry, the viral genome is translated on the rough endoplasmic reticulum (ER). The resulting HCV polyprotein is then cleaved by both viral and host factors to form 10 distinct proteins. Expression of HCV proteins causes major rearrangements of host cell membranes, leading to the formation of a complex membranous environment conducive to viral replication and assembly, termed the membranous web (MW). The virus-induced MW is essential for the viral replication cycle and harbours compartments that are physically separated from the surrounding cytosol [2, 3]. Host cell membrane rearrangements have been observed for all positive-strand RNA viruses and they can generally be characterized by the induction of two different membrane alterations: those containing double membrane vesicles, and those that form invaginated vesicles or spherules [4–19]. Replication complexes formed by several flaviviruses, including Dengue virus (DENV) and West Nile virus (WNV), contain ER-derived membrane sheets with numerous invaginated vesicles that maintain contact with the surrounding cytosol through narrow 11 nm pores located at the neck of the vesicle [11, 12]. By contrast, the HCV-induced MW is characterized by the clustering of single membrane vesicles and double membrane vesicles (DMVs) as well as multivesicular bodies, all within specific cytoplasmic regions that are also enriched for lipid droplets and ER membranes [8, 20–22]. Although the architecture and topology of the MW has been extensively studied, the spatial organization and function of its various membrane structures is still poorly understood. Several recent studies have proposed a prominent role for DMVs during HCV infection, by demonstrating that viral replication occurs in association with DMVs, and that these structures are vital for the viral life cycle [8, 23]. However, the precise role of DMVs in the viral life cycle and the spatial organization of different viral processes within the MW have not yet been described.

One proposed function for the MW is to conceal viral replication intermediates from cytoplasmic pattern recognition receptors (PRRs) and to limit host cell immune activation [9, 24]. Recognition of viral pathogen-associated molecular patterns (PAMPs), including double-stranded RNA (dsRNA), single-stranded RNA (ssRNA), and polyuridine signatures, is an important mechanism for immune activation in host cells. In virus-infected cells, PAMP recognition is accomplished primarily by Toll-like receptor 3 (TLR3) in endosomes or at the plasma membrane, and by RIG-I-like receptors (RLRs) in the cytosol. RLR’s include RIG-I and MDA5, which are cytoplasmic proteins that each contains two caspase-recruitment domains (CARDs) and a DExD/H-box helicase domain [25]. Both RIG-I and MDA5 interact with viral RNA molecules, with RIG-I preferentially recognizing 5’-phosphorylated blunt ends of viral genomic dsRNA and MDA5 binding long stretches of dsRNA [26–28]. Importantly, RIG-I also recognizes HCV ssRNA, binding to both the 5’ppp and the poly-U/UC region in the 3’ NTR [29]. Ligand recognition by either RIG-I or MDA5 results in activation of MAVS, nuclear translocation of IRF-3 and NF-κB, and transcriptional activation of early immune response genes [30–33].

RNA viruses have developed numerous strategies to block or avoid PRR initiated host responses. For example, both HCV and DENV proteases cleave critical molecules involved in the early stages of RLR signalling pathways, resulting in the abrogation of downstream signalling and immune activation [34, 35]. Additionally, tick-borne encephalitis virus has been proposed to evade RLR activation through the formation of intracellular membrane structures that sequester viral dsRNA away from the cellular recognition factors [36]. Immune evasion by hiding viral PAMPs from PRRs has also been postulated for a number of other viruses, but this has not yet been experimentally confirmed [24].

Previously, we have shown that components of the nuclear pore complex (NPC) redistribute to regions of HCV replication and assembly within the MW, and we provided evidence supporting a role for the nuclear transport machinery in the formation and function of the MW [37, 38]. Comprised of ~30 distinct proteins, termed nucleoporins (Nups), the NPC is a large macromolecular structure that facilitates bidirectional transport of macromolecules across the nuclear envelope between the cytoplasm and nucleoplasm. Transport through the NPC is mediated by soluble nuclear transport factors, many of which are members of a family of proteins termed karyopherins. Some karyopherins function in nuclear import (importins), while others export molecules from the nucleus (exportins). Cargos destined for the nucleoplasm contain short amino acid sequences termed nuclear localization signals (NLSs) that bind importins, while molecules exported from the nucleus contain nuclear export signals (NESs) that bind exportins. The resulting karyopherin-cargo complexes can then move through the NPC (reviewed in [39]).

Many viral proteins interact with components of the NPC or nuclear transport pathway (reviewed in [40, 41]). In most cases, these interactions reflect either a nuclear phase of the viral infection or a host-viral protein interaction that alters nuclear transport pathways. However, in the case of HCV, previous data indicate a cytoplasmic role for the nuclear transport machinery in infected cells [37]. In this work, a model was proposed in which NPC-like structures present within the MW facilitate transport between compartments within the MW and the surrounding cytosol in HCV-infected cells. This model predicts that NLS sequences found in certain proteins, including the viral proteins Core, NS2, NS3, and NS5A, as well as nuclear factors, are important for allowing access to the MW, while other proteins lacking NLSs, such as RLRs, are inhibited from entry [42, 43]. The use of NPC-mediated transport between cytoplasmic compartments in HCV-infected cells may constitute an important mechanism by which HCV evades RLR activation while maintaining active replication complexes.

In this study, we have investigated the organization of the MW and further evaluated the role of the nuclear transport machinery in the formation of a selective barrier between the viral replication/assembly complexes and the cytosol. We present data supporting a role for virus-induced cytoplasmic compartments in concealing viral PAMPs from RLR recognition in the cytoplasm of cells infected with positive-strand RNA viruses. We also provide evidence for the formation of distinct replication and assembly compartments within the MW, and we describe a useful technique for isolating these compartments. Furthermore, we present data indicating the nuclear transport machinery contributes to the selective movement of molecules into the MW and the exclusion of cytoplasmic RLRs.

## Results

### HCV infection induces the production of cytoplasmic compartments lacking RLRs

Several studies have reported that the MW appears to protect viral proteins and RNA from exogenously added nucleases [2, 3, 36]. This compartmentalization is also supported by our observation that HCV proteins present in the MW appear in regions of the cytoplasmic largely devoid of microtubules [37]. Importantly, the barrier formed by the membrane structures of the MW appears to be selectively permeable, and the nuclear transport machinery contributes to controlling access of proteins to compartments within the MW [37, 38]. For example, GFP reporter proteins containing NLS sequences can access regions within the MW containing HCV proteins [37, 38].

**Fig 1:**
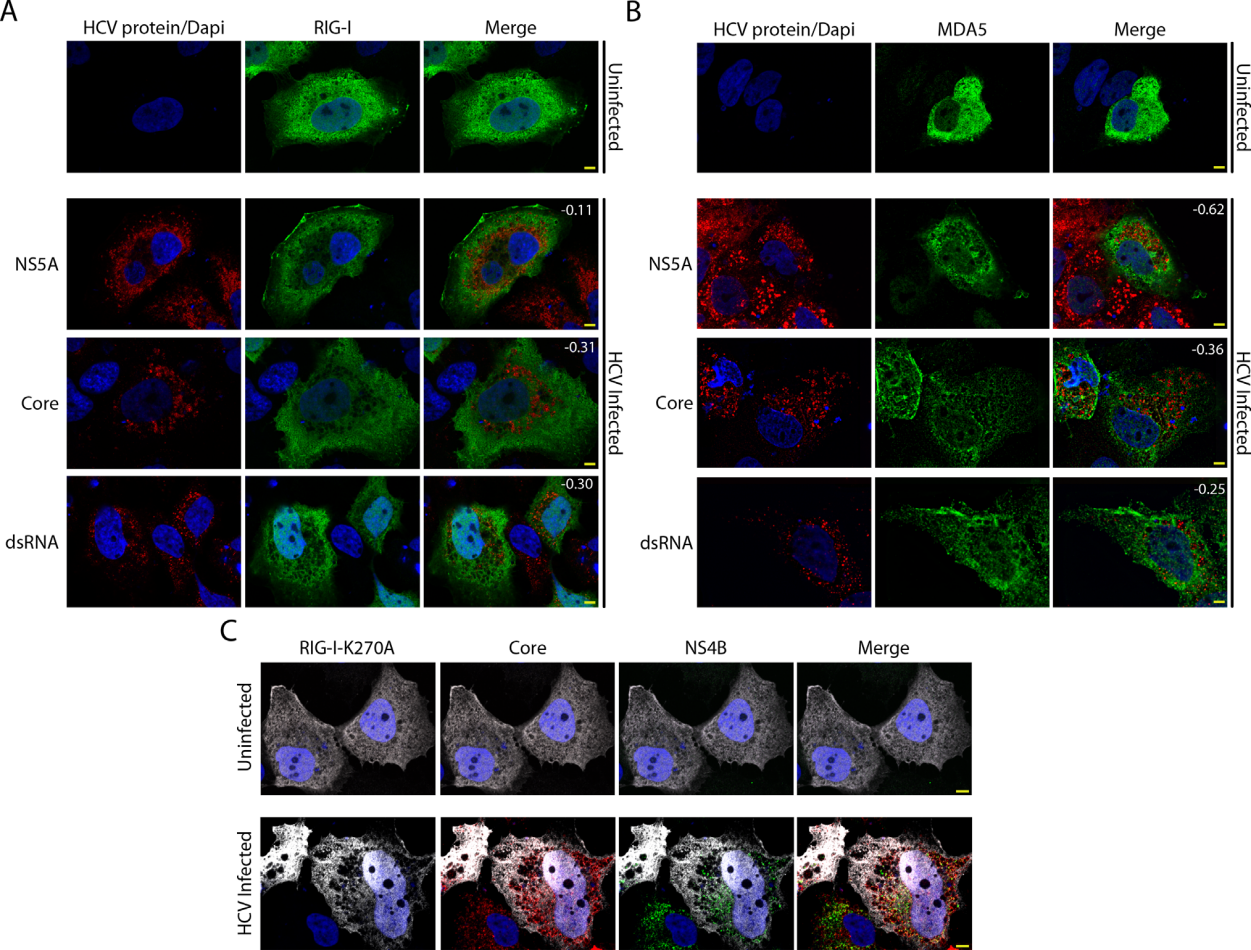
Exclusion of RLRs from cytoplasmic regions enriched for HCV proteins. Uninfected and HCV-infected Huh7.5 cells were transfected with constructs encoding for FLAG-tagged RIG-I (A), V5-tagged MDA5 (B), or FLAG-tagged RIG-I-K270A (C) 2 days after HCV infection. On day 4 after infection, the localization of HCV proteins and RLRs in uninfected and HCV-infected cells was evaluated by indirect immunofluorescence confocal microscopy using antibodies specific for the indicated HCV protein or double-strand viral RNA and either the V5 or FLAG epitope tag. DNA was detected with DAPI (blue) and scale bars represent 5 μm. Pearson’s correlation coefficients shown in the merge images of HCV-infected cells in panels A and B were calculated using Coloc2 software in ImageJ and represent overlap between the red and green fluorescent channels in the indicated image.

Active exclusion of proteins from the MW could represent an innate immune evasion strategy employed by the virus [8, 24, 37]. To test this hypothesis, we transfected uninfected or HCV-infected cells with constructs encoding FLAG-tagged RIG-I or V5-tagged MDA5 and visualized the cellular distribution of these RLRs by confocal immunofluorescence microscopy (Fig 1). In uninfected cells, we observed a largely diffuse cytoplasmic localization for both RIG-I and MDA5, with small areas devoid of signal likely arising from the exclusion of the RLRs from vesicles (Fig 1A and 1B). In HCV-infected cells, RIG-I and MDA5 were absent from larger regions of the cytoplasm and, importantly, these areas contained the majority of HCV proteins examined and viral dsRNA (Fig 1A and 1B bottom panels). Pearson’s correlation coefficients showed that there was a negative correlation between fluorescent signals observed for the HCV proteins/RNA and either RIG-I or MDA5, implying that the RLRs are restricted from accessing regions of the cytoplasm occupied by the MW. To further evaluate this apparent segregation, we examined the subcellular localization of the MDA5 reporter in Huh7 cells harbouring the JFH-1 subgenomic replicon. In replicon cells transfected with constructs coding for MDA5, we observed that MDA5 and viral proteins were also visible in distinct, largely non-overlapping regions of the cytoplasm similar to that seen in the HCV-infected cells (S1A Fig). These observations support the hypothesis that viral compartments inhibit entry of RLRs.

A caveat of the experiments examining the exogenous expression of RLRs is that overexpression of the active form of RIG-I in HCV-infected Huh7.5 cells leads to immune activation, which impedes viral replication and could alter the structure of the MW [29]. To avoid this complication, we used a point mutant of RIG-I (RIG-I-K270A) that can still bind to viral RNA but lacks helicase activity necessary for tracking along the RNA strand and downstream immune signalling [44]. We observed that the subcellular localization of Rig-I-K270A was similar to that of the RIG-I in uninfected and HCV-infected cells (Fig 1C and S1B Fig). Furthermore, RIG-I-K270A was also reduced in regions of the cytoplasm where HCV NS4B and core were concentrated (Fig 1C). Rig-I-K270A signal was also absent from cytoplasmic regions containing lipid droplets, which are induced by HCV infection and generally associated with core protein, as well as from regions surrounding lipid droplets (S1C Fig).

### HAV infection induces cytoplasmic compartments lacking RIG-I

**Fig 2:**
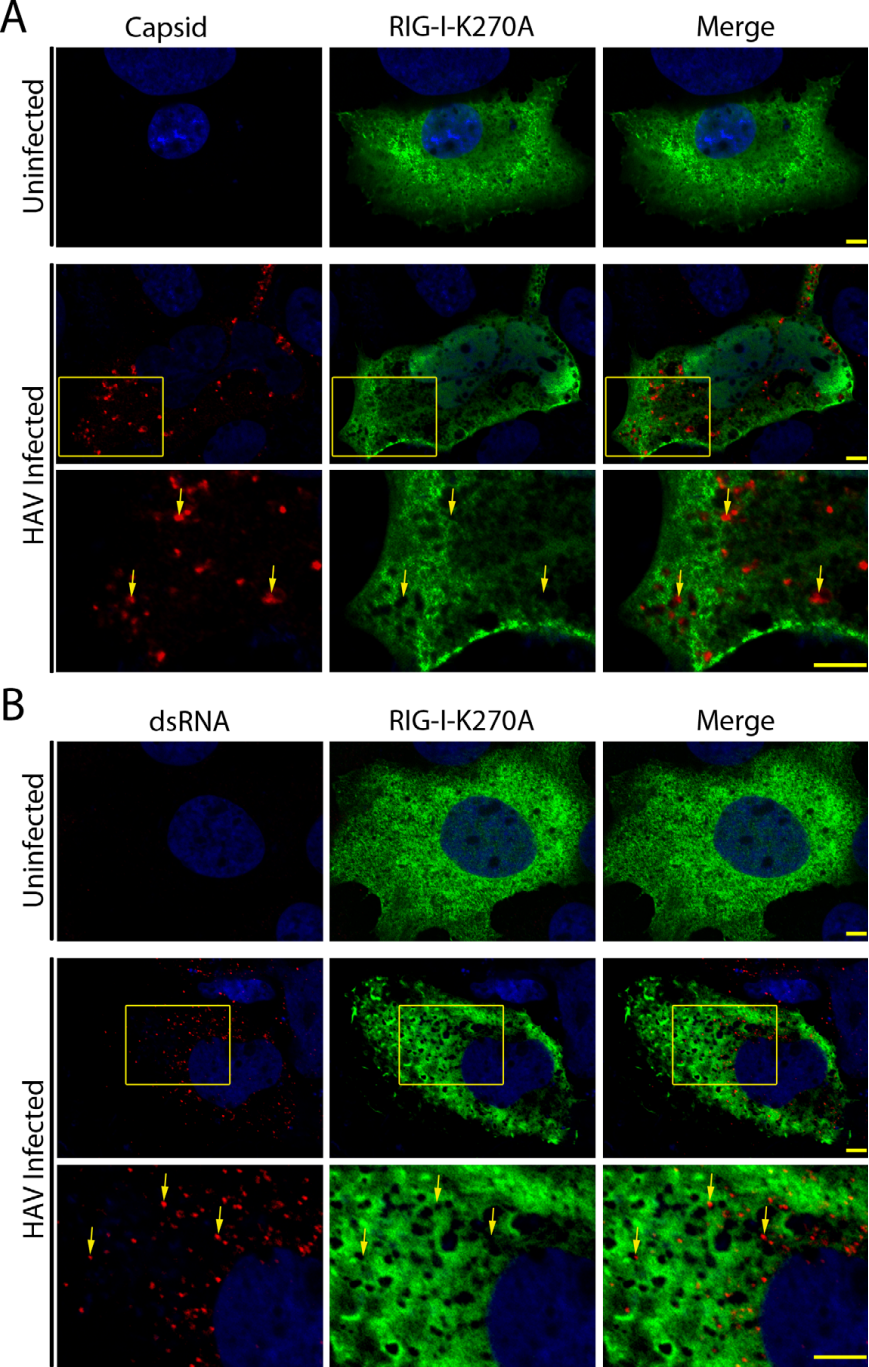
Exclusion of RLRs from cytoplasmic regions enriched for HAV proteins. A and B) Uninfected and HAV-infected Huh7.5 cells were transfected 5 days after viral infection with constructs encoding the catalytically inactive FLAG-tagged RIG-I-K270A. On day 7 after HAV infection, the localization of RIG-I-K270A (green) and either HAV capsid protein (red, panel A) or dsRNA (red, panel B) were evaluated by indirect immunofluorescence confocal microscopy using specific antibodies. DNA was stained with DAPI (blue) and scale bars represent 5 μm. Arrows point to regions of the cytoplasm that contain capsid proteins or dsRNA but lack RIG-I-K270A signal and the boxes in the middle panels represent the area of magnification in the bottom panels.

All positive-strand RNA viruses reorganize host cell membranes, often forming distinct replication complexes [9, 45]. To test if the exclusion of RLRs from regions occupied by viral replication compartments is a phenomenon displayed by other positive-strand RNA viruses, we examined the localization of RIG-I-K270A in hepatitis A virus (HAV)-infected cells. As shown in Fig 2, in HAV-infected Huh7.5 cells, we observed exclusion of RIG-I-K270A from cytoplasmic regions containing HAV capsid protein or viral dsRNA, results similar to that seen in HCV-infected cells. These data are consistent with the proposed idea that virus-induced cytoplasmic compartments may represent a common immune evasion strategy of positive-strand RNA viruses.

### RLRs reside primarily in cytoplasmic regions distinct from of viral replication and assembly

**Fig 3:**
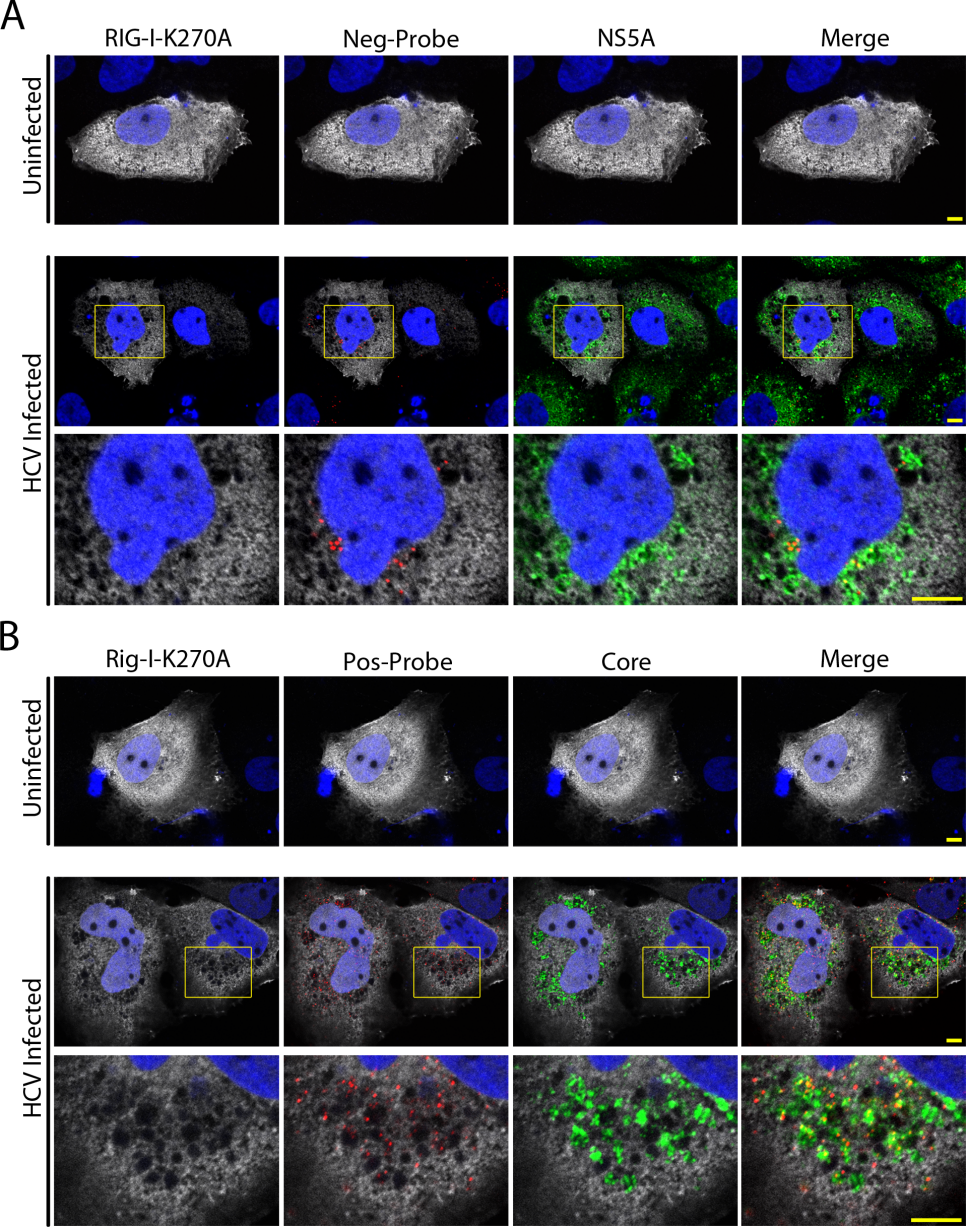
Exclusion of RIG-I from compartments containing plus-strand and minus-strand HCV RNA. A and B) Uninfected and HCV-infected Huh7.5 cells were transfected with constructs encoding for FLAG-tagged RIG-I-K207A 2 days after HCV infection. On day 4 after infection, cells were probed with antibodies directed against the FLAG epitope (grey) and either HCV core or NS5A (green). DNA probes (Affymetrix) targeted to either the positive-strand or the negative-strand of the HCV RNA (red) were then hybridized to the viral RNA. DNA was stained with DAPI (blue) and cells were examined by confocal fluorescence microscopy. Boxed regions in the middle row of both panels outline the area of magnification presented in the bottom rows. Scale bars represent 5 μm.

The results presented above imply that HCV infection induces the formation of the MW to sequester viral processes from RLRs found in the surrounding regions of the cytoplasm, a condition that would limit the innate immune response. In positive-strand RNA virus infection, viral RNA is involved is several different processes including genome replication, translation, and virion assembly. To better understand the spatial relationship of these processes relative to the MW and RLRs, we used fluorescence microscopy to examine the localization of viral RNA and viral proteins, and we compared these to ectopically expressed RLRs. The localization of viral RNAs was detected using branched DNA probes directed against either the positive-strand or negative-strand of the HCV genome [46–48]. This methodology has previously been used for single molecule detection of host cell mRNA transcripts, as well as for detection of viral RNAs [48–51]. For our studies, cells harbouring the FLAG-tagged RIG-I-K270A construct were co-stained with antibodies directed against the FLAG epitope and either HCV core or NS5A. The cells were then examined by in situ hybridization using positive-strand or negative-strand RNA probes (Fig 3 and S2 Fig). Manders Overlap Coefficients were used to determine the percent overlap of positive- or negative-strand HCV RNA with immunofluorescence signals produced by antibodies against NS5A, RIG-I-K270A, or core. In all cases, percent overlap is calculated from ~15 cells. In cells analyzed for the localization of NS5A, RIG-I-K270A, and negative-strand HCV RNA, we observed that the majority of the negative-strand viral RNA (82% of the fluorescence signal) overlapped with NS5A signal. Moreover, regions containing negative-strand RNA lacked RIG-I, as only 16% fluorescence signal from the negative-strand probe overlapped with the RIG-I signal (Fig 3A). We infer from these data that RIG-I is present in regions of the cytoplasm largely distinct from negative-strand viral RNA containing replication centers within the MW. Similarly, in HCV-infected cells co-stained for core protein, RIG-I-K270A, and positive-strand RNA, both positive-strand RNA and core protein were predominantly localized to the same MW regions, with 55% of the positive-strand RNA fluorescence signal overlapping with the core protein signal. By contrast, only 28% of positive-strand RNA signal overlapped with the RIG-I signal (Fig 3B). The presence of a less abundant pool of positive-strand RNA (28% of the fluorescent signal) in regions that overlapped with the RIG-I signal implies that although the majority of positive-strand HCV RNA is present within MW compartments that lack RIG-I, another pool exists outside these regions, which is consistent with the need for translation of viral RNA.

### Cell fractionation reveals distinct compartments enriched for genome replication and viral assembly factors

Our localization studies detect the presence of viral RNA in distinct locations in the cell, primarily within the MW but also in the surrounding cytoplasm. This distribution is consistent with the various pathways in which both positive- and negative-strand viral RNA are used, including genome replication, translation, and viral assembly, and the proposed spatial separation for these processes in cells infected with HCV or various other positive-strand RNA viruses [11, 24, 52]. To further evaluate the distribution of key HCV proteins and RNA in cells, we have used subcellular fractionation to isolate various membrane fractions from infected cells. The bulk of HCV proteins examined were contained within two cytoplasmic membrane fractions. One lighter membrane fraction contains all the cytoplasmic membranes that did not sediment into a mitochondrial pellet fraction, including ER-derived microsomal membranes, which we term the microsomal fraction. The second contains membranes associated with the mitochondrial pellet including a mitochondrial-associated membranes (MAMs) and cytoplasmic membranes with similar sedimentation and buoyant density characteristics. We will refer to this fraction as the MAM fraction. In HCV-infected cells, both of the ‘microsomal’ and ‘MAM’ fractions are predicted to contain membranes with unique characteristics arising during the HCV-induced changes in cellular membrane structure.

**Fig 4:**
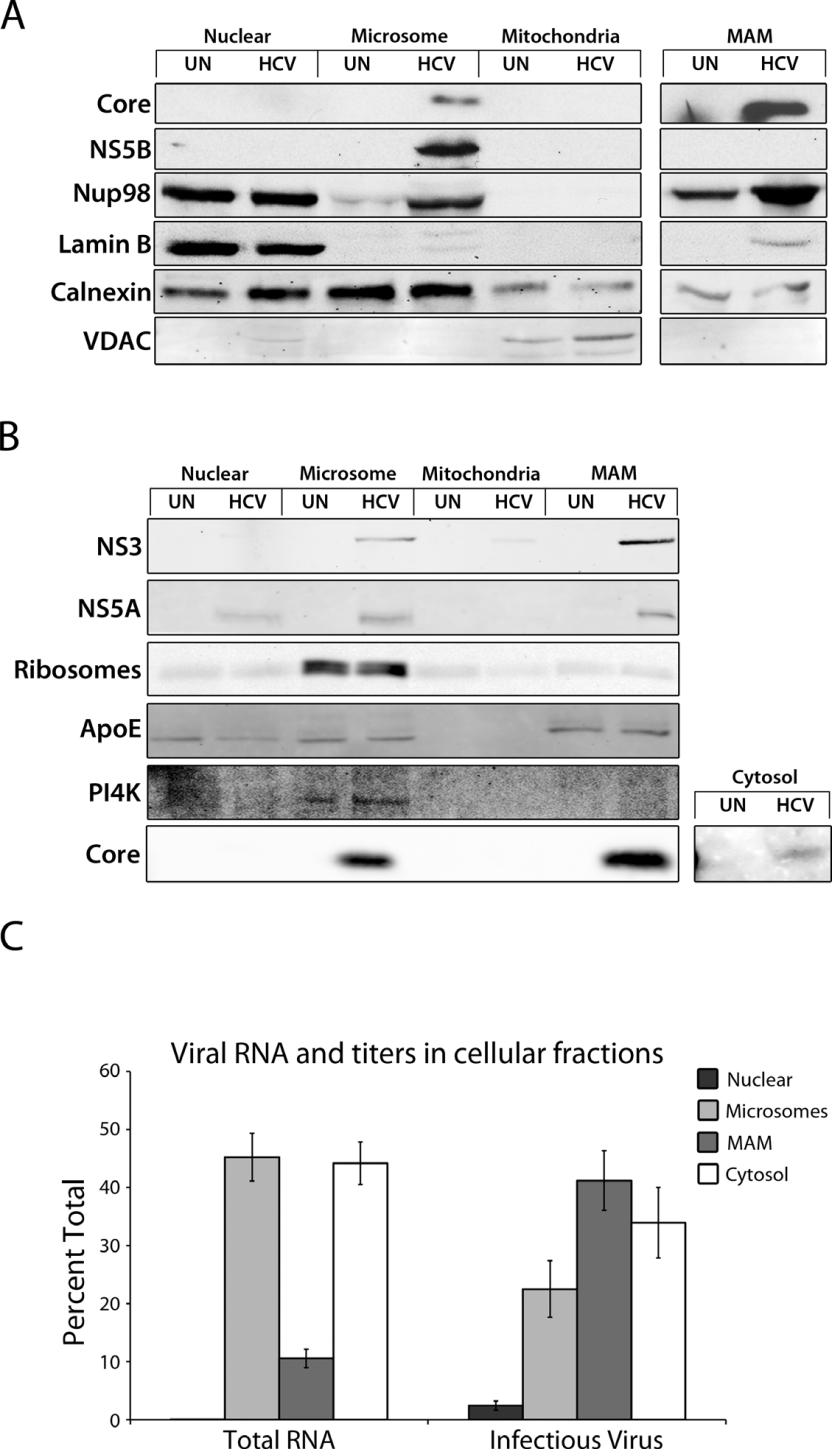
HCV proteins are associated with physically distinct membrane fractions in infected cells. A-C) Total cell lysates isolated from uninfected (UN) or HCV-infected (HCV) Huh7.5 cells were subjected to subcellular fractionation. A and B) Western blotting with antibodies specific for the indicated proteins was used to evaluate their relative amounts in various subcellular membrane fractions. Indicated above the blot image are membrane species predicted to reside in the fractions analyzed. Equal amounts of total protein were loaded into each lane. All samples were run on the same gel and images shown are derived from the same membrane. For the core cytosolic blot (B, bottom right) exposure levels were increased 4 fold in order to observe the signal. C) The total HCV RNA present in the nuclear, microsomal, MAM, or cytosolic fractions analyzed in panel A was determined by qPCR. The number of infectious HCV particles in these same fractions was determined by infecting Huh7.5 cells with a portion of the sample from each fraction followed by counting focus-forming units identified using indirect immunofluorescence microscopy and antibodies directed against HCV core protein.

We observed an enrichment of viral polymerase NS5B in the microsomal membrane fractions, but only low levels of core and NS3. By contrast, core and NS3, but not NS5B, were enriched in the MAM fraction (Fig 4A and 4B). NS5A was observed at similar levels in both the microsomal and MAM fractions (Fig 4B). Thus, key components of viral replication were enriched within microsomal membrane fractions, while assembly center components were found in the MAM fraction. We also evaluated the levels of total viral RNA and infections virus present in the various membrane fractions and cytosol (Fig 4C). Viral RNA and infectious virus were detected in the cytosol, presumably arising from membrane fragmentation that occurs during cell lysis. Within the membrane fractions, we observed that higher levels of total viral RNA were present in the microsomal fraction. However, approximately double the percentage of infectious virus was present in the MAM fraction. These fractionation results lead us to conclude that distinct replication and assembly compartments are present in HCV-infected cells, with the microsomal membrane fraction enriched for viral replication complexes (NS5B and viral RNA) and the MAM membrane fraction enriched for viral assembly complexes (Core, NS3, and infections virus). Moreover, the lower percentage of viral RNA (~10% of total RNA) found in the MAM fraction with viral assembly components implies that the majority of the membrane-associated viral RNA within the cell is likely engaged in replication or translation rather than virion assembly.

The separation of virus replication and assembly implies that host proteins required for these processes are present in in the microsomal and MAM fractions. We examined the location of several host proteins known to function during either viral replication or virion assembly. Previous studies have implicated Apolipoprotien E (ApoE) in viral assembly and 1-phosphatidylinositol 4-kinase (PI4K) in viral replication [53–56]. Therefore, we probed membrane fractions derived from infected cells with antibodies directed against ApoE and PI4K (Fig 4B). As shown in Fig 4C, ApoE is present in the MAM fraction, which is consistent with the conclusion that this fraction is enriched for viral assembly complexes. Additionally, we show that PI4K is primarily found in the microsomal fraction. These observations are consistent with the microsomal fraction being enriched for viral replication complexes.

### Ribosomes are largely excluded from the MW

**Fig 5:**
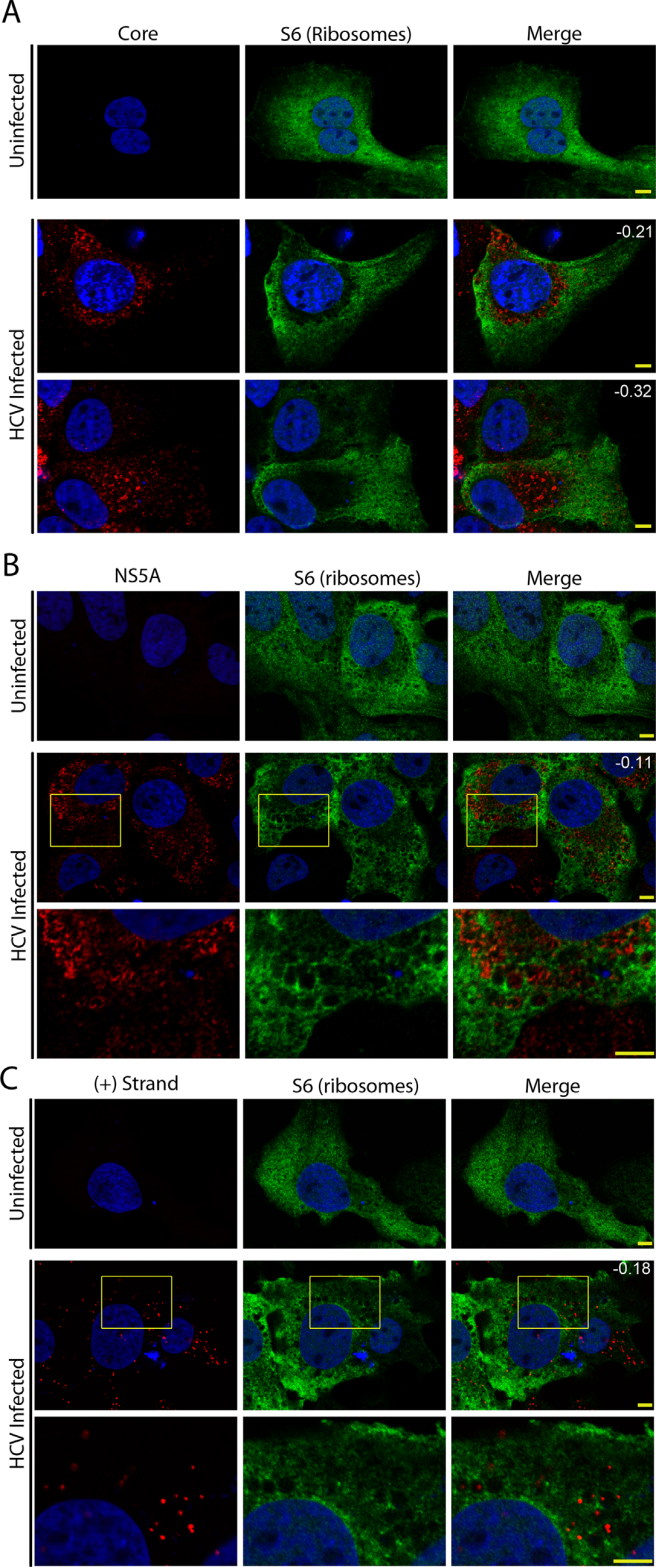
Exclusion of ribosomes from viral replication compartments. A) Huh7.5 cells were untreated or infected with HCV for four days. The localization of the 40S ribosomal subunit protein S6 (green), HCV core protein (panel A, red), HCV NS5A protein (panel B, red), or positive-strand HCV RNA (panel C, red) in cells was evaluated by indirect immunofluorescence confocal microscopy using specific antibodies. DNA was stained with DAPI (blue). Pearson’s correlation coefficients shown in the merge images of HCV-infected cells were calculated using Coloc2 software in ImageJ and represent overlap between the red and green fluorescent channels in the indicated image. For panels B and C, the boxed regions in the middle row of both panels outline the area of magnification presented in the bottom rows. Scale bars represent 5 μm.

Results obtained from the subcellular fraction of HCV-infected cells show that the majority of membrane-bound viral RNA is associated with the microsomal fraction, and the concentration of NS5B in this fraction suggests this RNA fraction includes replication complexes. These data are consistent with previous studies reporting that positive-strand RNA virus replication complexes are formed in association with ER-like membranes (Reviewed in [57]). The microsomal fractions are also predicted to contain positive-strand RNA being translated into HCV polyprotein on membrane-bound ribosomes [6, 11–13]. However, the processes of viral RNA replication and translation have been suggested to occur in distinct locations [48, 57, 58]. To further assess the spatial relationship between translation and the MW, we examined the distribution of ribosomes in HCV-infected cells using immunofluorescence microscopy. We stained uninfected or HCV-infected Huh7.5 cells with antibodies directed against the S6 protein component of the 40S ribosomal subunit. In uninfected cells, ribosomes were distributed throughout the cytoplasm (Fig 5A). However, in infected cells the vast majority of the S6 protein was excluded from regions of the cytoplasm containing the bulk of the HCV core protein (Fig 5A). The separation of the two fluorescent signals was also supported by negative Pearson’s correlation coefficients (Fig 5). The same general phenotype was observed when we compared the localization of the S6 protein to that of NS5A or positive-strand HCV RNA (Fig 5B and 5C), consistent with the conclusion that the bulk of the ribosomes are located in regions of the cell separate from HCV replication and assembly complexes. Consistent with our observations in Fig 3B showing that a portion of the viral positive-strand RNA colocalizes with RIG-I, Manders Overlap Coefficient’s revealed that, while the majority of the positive-strand RNA (76% of the fluorescent signal) was found in regions lacking ribosomal signal, a smaller pool of positive-strand RNA (24% of the fluorescent signal) overlapped with the ribosomal S6 signal (Fig 5C). To determine whether the signal observed for ribosomal proteins correlated with that of RIG-I, cells were transfected with constructs encoding RIG-I-K270A followed by staining with antibodies directed against the FLAG epitope, S6 ribosomal protein, and HCV core. In both uninfected and HCV-infected cells, there was significant overlap observed between RIG-I-K270A and the S6 protein (S3 Fig). This spatial separation between the MW and the bulk of the ribosomal proteins is consistent with a model in which translation of the viral polyprotein occurs outside of the MW.

### Addition of a nuclear localization signal (NLS) to RLRs allows access into the MW

**Fig 6:**
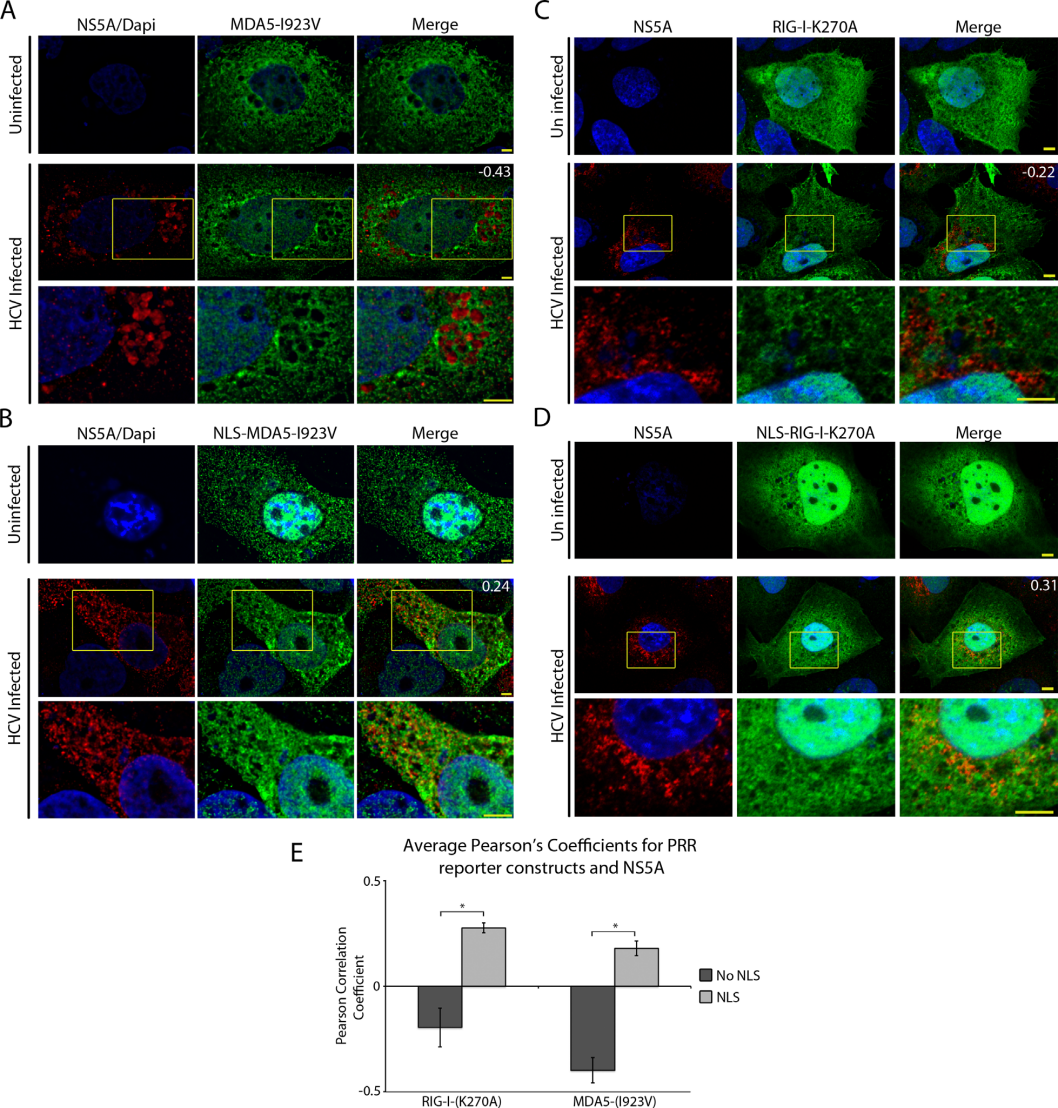
NLS-tagged RIG-I and MDA5 colocalize with HCV proteins. A and B) Uninfected and HCV-infected Huh7.5 cells were transfected with constructs encoding either V5-tagged MDA5-I923V (A) or V5-tagged NLS-MDA5-I923V (B) 2 days after HCV infection. On day 4 after infection, the localization of HCV NS5A protein (red) was compared to that of MDA5-I923V or NLS-MDA5-I923V (green) using antibodies specific for the HCV NS5A and the V5 epitope tag and indirect immunofluorescence microscopy. DNA was stained with DAPI (blue). Boxed regions in the middle row of both panels outline the area of magnification presented in the bottom rows. Scale bars represent 5 μm. C and D) Uninfected or HCV-infected Huh7.5 cells were transfected with constructs encoding for either GFP-tagged RIG-I-K270A (C) or GFP-tagged NLS-RIG-I-K270A (D) 2 days after HCV infection. On day 4 after infection, the localization of HCV NS5A protein (red) was compared to that of GFP-tagged RIG-I-K270A or NLS-RIG-I-K270A (green). NS5A was detected using specific antibodies and indirect immunofluorescence and the GFP fusions were visualized directly using fluorescence confocal microscopy. DNA was stained with DAPI (blue). Boxed regions in the middle row of both panels outline the area of magnification presented in the bottom rows. Scale bars represent 5 μm. For all image panels, Pearson’s correlation coefficients shown in the merge images of HCV-infected cells were calculated using Coloc2 software in ImageJ and represent overlap between the red and green fluorescent channels in the indicated image. E) Pearson’s correlation coefficients were determined to assess overlap of HCV NS5SA and either GFP or V5 fluorescence signals in confocal sections derived from HCV-infected cells expressing GFP-tagged RIG-I-K270A, GFP-tagged NLS-RIG-I-K270A, V5-tagged MDA5-I923V or V5-tagged NLS-MDA5-I923V. The values presented represent an average over 10 images (> 15 cells) and the error bars indicate standard error. Significance was evaluated using t-tests comparing and p-values less than 0.05 (*) are indicated.

Our results indicate that the HCV-induced MW segregates activities associated with this structure, such as HCV replication and viral assembly, from cytosolic factors including ribosomes and RLRs. We have previously proposed that access of certain macromolecules to compartments within the MW is regulated by NPCs and nuclear transport factors, an observation consistent with both the presences of nuclear transport signals in multiple HCV proteins and our previous results showing a GFP-NLS-tagged reporter protein could enter regions of the cytoplasm occupied by the MW [37, 38] (see S4 Fig). On the basis of these data, we hypothesized that placing an NLS on an RLR would overcome the selective barrier between the cytoplasm and the MW [37, 38]. For these experiments, an extensively studied NLS derived from the SV40 large T antigen (here referred to simply as the NLS) was used in the construction of various fusion proteins [59]. Constructs encoding GFP-tagged RIG-I-K270A, GFP-tagged NLS-RIG-I-K270A, V5-tagged MDA5-I923V, or V5-tagged NLS-MDA5-I923V were transfected into uninfected or HCV-infected Huh7.5 cells, and the localization of each protein was compared to that of HCV NS5A. Similar to the K270A mutation in RIG-I, the I923V mutation in MDA5 inhibits MDA5-mediated activation of immune signalling pathways [60]. Consistent with our observations of MDA5 (Fig 1B), the MDA5-I923V mutant was also observed outside cellular regions containing NS5A (Fig 6A). In uninfected cells, both NLS-MDA5-I923V and NLS-RIG-I-K270A exhibited an increased nuclear signal over those not containing an NLS sequence (MDA5-I923V and RIG-I-K270A), demonstrating that the NLS sequence is functional (Fig 6A–6D). Strikingly, when the NLS-MDA5-I923V and NLS-RIG-I-K270A proteins were examined in HCV-infected cells, the level of overlap between these RLRs and NS5A significantly increased compared to RLRs lacking the NLS (Fig 6A–6D). Quantification of signal overlap using average Pearson’s correlation coefficients of ~20 cells revealed a significant increase in overlap between the fluorescent signals associated with NS5A and NLS-tagged RLRs when compared to NS5A and the untagged RLRs (Fig 6E). These results led us to conclude that the addition of an NLS to RLRs, allowed the fusion protein to overcome the exclusion barrier normally preventing RLRs from entering the MW.

**Fig 7:**
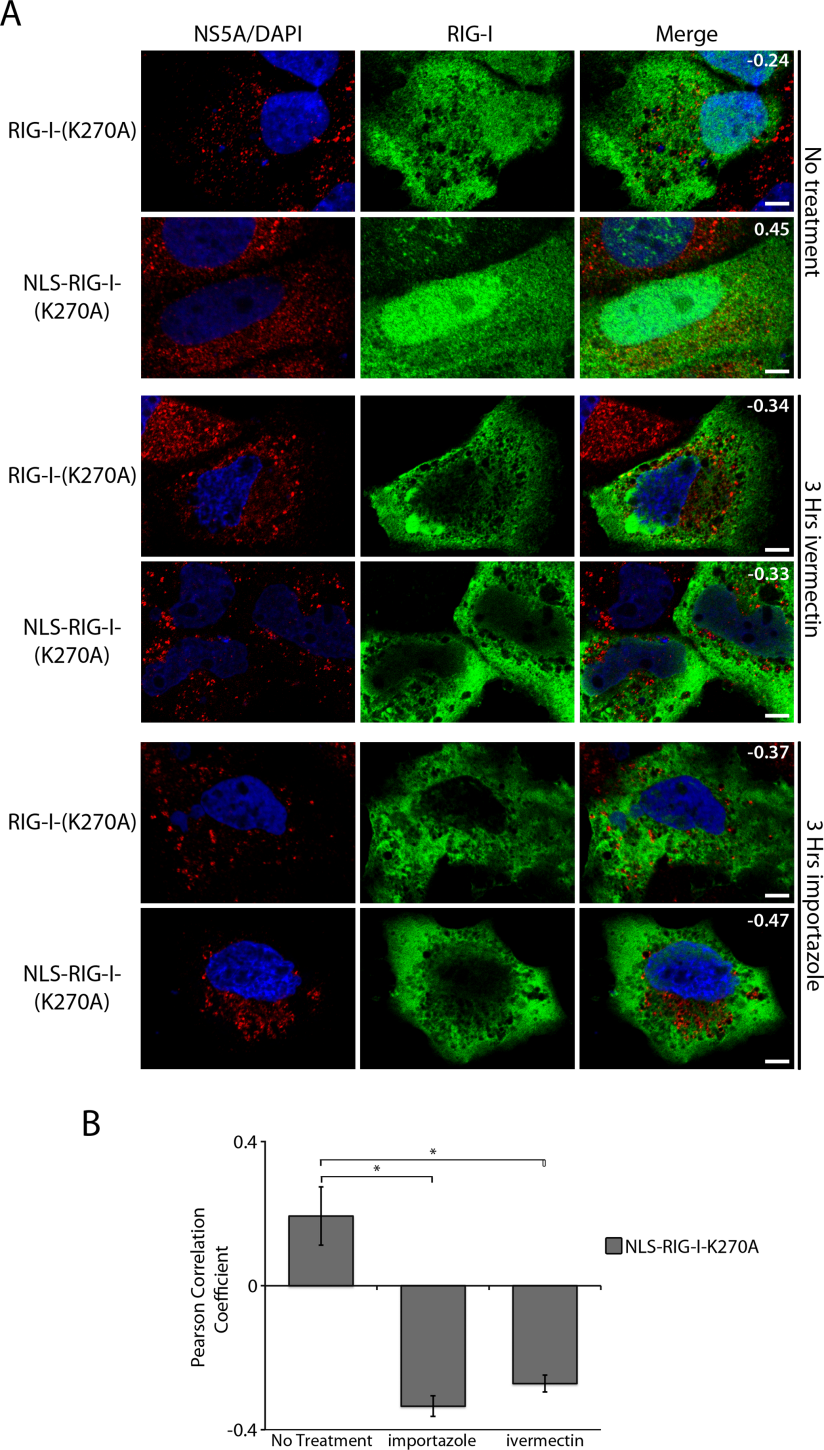
Inhibition of NLS-importin α/β transport limit access of NLS-tagged RIG-I to the MW. A and B) HCV-infected Huh7.5 cells were transfected with constructs encoding for either GFP-tagged RIG-I-K270A or GFP-tagged NLS-RIG-I-K270A 2 days after HCV infection. On day 4 after infection, cells were treated with 25 μM ivermectin or 40 μM importazole for 4 hours and then examined. A) HCV NS5A was detected using specific antibodies and indirect immunofluorescence and the GFP fusions were visualized directly using fluorescence confocal microscopy. DNA was stained with DAPI (blue). Boxed regions in the middle row of both panels outline the area of magnification presented in the bottom rows. Scale bars represent 5 μm. Pearson’s correlation coefficients shown in the merge images of HCV-infected cells were calculated using Coloc2 software in ImageJ and represent overlap between the red and green fluorescent channels in the indicated image. B) Pearson’s correlation coefficients were determined to assess overlap of HCV NS5SA and GFP signals in confocal images derived from HCV-infected cells expressing the indicated RIG construct. The values presented represent an average over 10 images (at least 15 cells) and the error bars indicate standard error. Significance was evaluated using t-tests and p-values less than 0.05 (*) are indicated.

These data are consistent with previous studies documenting a role for the nuclear transport machinery in controlling access of molecules to HCV-induced MW [37]. We evaluated this further by examining whether the ability of the NLS-RIG-I-K270A construct to access regions occupied by the MW required nuclear transport factors. For this analysis, we examined the effects of two small molecule inhibitors, ivermectin and importazole [61, 62], that specifically block the transport function of the SV40 large T antigen NLS cognate nuclear import receptor, the importin α/β dimer. HCV-infected cells expressing GFP-tagged RIG-I-K270A or GFP-tagged NLS-RIG-I-K270A were treated with either ivermectin or importazole for 3 hours and then evaluated by confocal microscopy. In the absence of the inhibitors, the degree of co-localization between RIG-I-K270A or NLS-RIG-I-K270A and NS5A in HCV-infected cells was similar to that presented Figs 1 and 6 (Fig 7 top panels), with the NLS-RIG-I-K270A construct and NS5A showing a positive Pearson correlation coefficient. However, when these cells were treated with ivermectin or importazole, the overlap between NLS-tagged RIG-I-I-K270A and NS5A was significantly decreased and a negative Pearson correlation coefficient was observed. By contrast, neither drug affected the distribution or the degree of overlap between RIG-I-K270A and NS5A (Fig 7). These results further support a model in which the nuclear transport machinery regulates access of proteins to regions within the MW.

### NLS-tagged RLRs stimulate immune responses in HCV-infected cells and inhibit HCV

**Fig 8:**
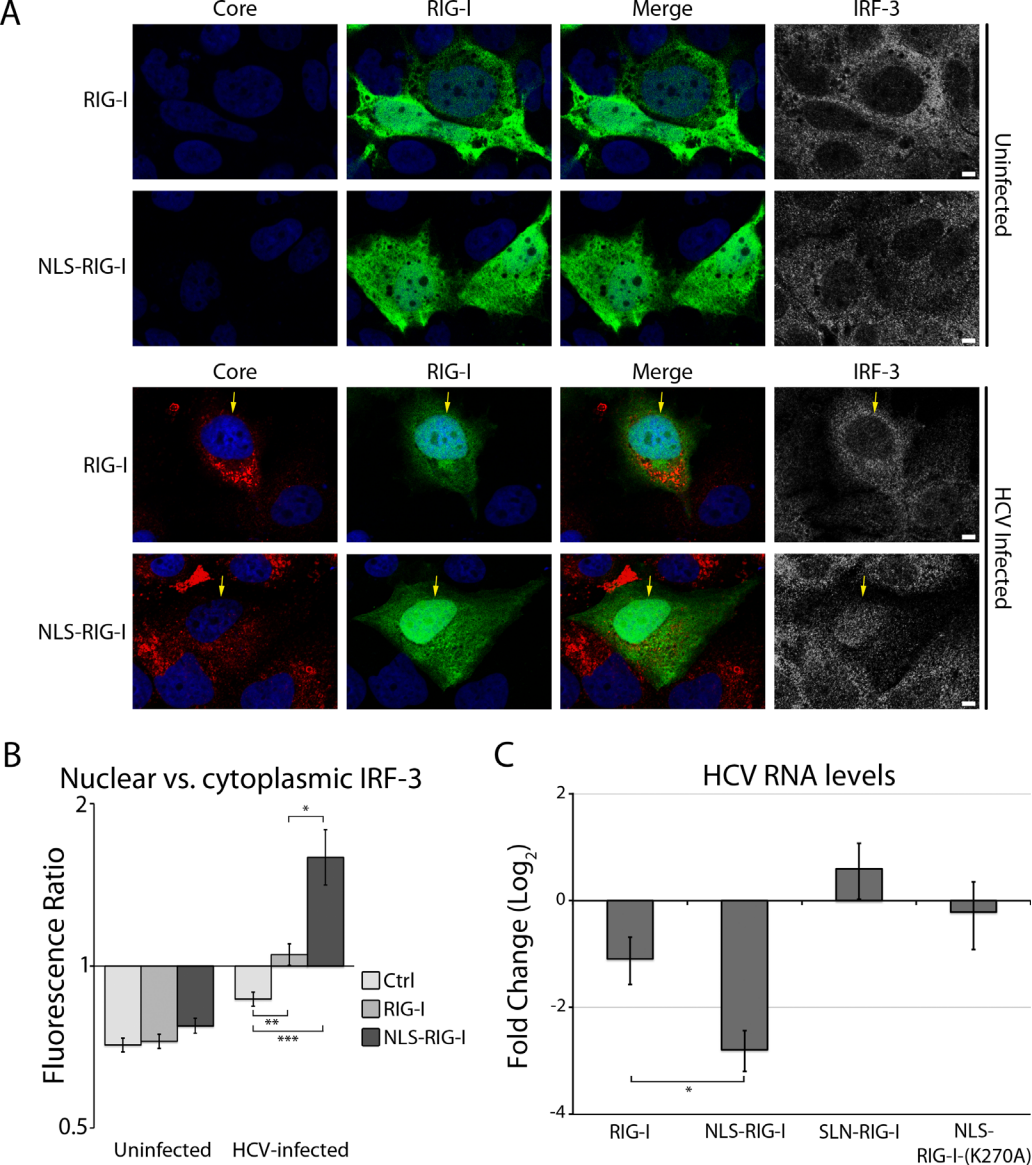
NLS-tagged RIG-I stimulates immune responses and decreased viral RNA levels in HCV-infected cells. A and B) Uninfected or HCV-infected Huh7.5 cells were transfected with constructs encoding either GFP-tagged RIG-I or GFP-tagged NLS-RIG-I 2 days after HCV infection. On day 4 after infection, the localization of IRF-3 (grey) was examined using specific antibodies and indirect immunofluorescence and the GFP fusions (green) were visualized directly using fluorescence confocal microscopy. In addition, to identify HCV-infected cells amongst those expressing the RIG-I constructs, cells were stained with antibodies directed against the HCV core protein (red). DNA was stained with DAPI (blue) and scale bars represent 5 μm. B) The ratio of nuclear to cytoplasmic fluorescence signal arising from IRF-3 indirect immunofluorescence was calculated using ImageJ for > 30 uninfected or HCV-infected cells alone or expressing either GFP-tagged RIG-I or GFP-tagged NLS-RIG-I. Significance was evaluated using t-tests and p-values less than 0.05 (*), 0.01 (**) or 0.001 (*) are indicated. C) Uninfected and HCV-infected Huh7.5 cells were transfected with constructs encoding for GFP-RIG-I, GFP-NLS-RIG-I, GFP-SLN-RIG-I, or GFP-NLS-RIG-I-K270A 1 day after HCV infection. 3 days after infection, cells were harvested and levels of intracellular HCV RNA were determined by qPCR. Fold change of intracellular HCV RNA was calculated relative to HCV-infected cells transfected with constructs encoding for GFP alone.

We postulate that exclusion of RLRs from the MW contributes to masking the viral genome from the cell’s innate immune response. Since our data show that adding an NLS to the RLRs allows the fusion proteins to use the nuclear transport machinery and access the MW, we examined the effects of NLS-RLR entry into the MW on innate immune activation in HCV-infected cells. To assess immune response, we examined the localization of IRF-3 in uninfected or HCV-infected cells expressing either RIG-I or NLS-RIG-I (Fig 8A and 8B). The re-localization of IRF-3 to the nucleus is an initial step in RLR-mediated immune activation [63]. Therefore, we used immunofluorescence microscopy to monitor nuclear translocation of IRF-3 to determine immune activation in cells expressing the RIG-I constructs. This approach allowed us to assess activation of the immune response in individual cells determined to be both HCV-infected or uninfected and transfected with the RIG-I fusion constructs. In uninfected cells, we found that the expression of RIG-I or NLS-RIG-I did not significantly alter the localization of IRF-3 compared to that of untransfected cells, with IRF-3 appearing largely cytoplasmic and excluded from the nucleus (Fig 8A and 8B). However, in HCV-infected cells, the nuclear localization of IRF-3 was significantly increased in cells expressing NLS-RIG-I as compared to those expressing RIG-I or untransfected cells (Fig 8A and 8B). We conclude from these results that the expression of NLS-RIG-I increases immune activation in HCV-infected cells to a greater extent than overexpression of wild-type RIG-I.

We also examined the effect of expressing various RIG-I constructs, including RIG-I, NLS-RIG-I, NLS-RIG-I-K270A, or SLN-RIG-I (SLN encodes a reverse sequence, non-functional NLS) on virus production. Similar levels of each of these constructs were detected in transfected cell populations (S5A Fig). Furthermore, the expression of each RIG-I construct alone stimulated only minimal, statistically insignificant, changes in the levels of immune stimulated genes compared to those expressing wild type RIG-I, indicating that the NLS tag has little or no effect on RIG-I-mediated immune activation (S5B Fig). We then tested the consequences of expressing these various RIG-I constructs in HCV-infected cells. For this analysis, cells were infected with HCV followed by transfection with each of the four RIG-I constructs, and total viral RNA levels were determined by real-time PCR. As predicted, increased cellular levels of RIG-I caused a small, but significant decrease in HCV RNA levels (Fig 8C). Strikingly, introduction of the NLS-RIG-I construct lead to significant further decrease in levels of HCV RNA as compared to wild-type RIG-I. By contrast, no changes were observed in cells transfected with the SLN-RIG-I or NLS-RIG-I-K270A expression constructs. These results further implicate the nuclear transport machinery as a regulator of access to sites of virus replication and assembly.

## Discussion

Much like other membrane-bound organelles, the membrane structures induced by positive-strand RNA viruses serve to both concentrate proteins within a specific area, thereby increasing efficiency of certain processes, and to spatially separate competitive reactions. A proposed function of sequestering viral replication complexes away from the surrounding cytosol is the concealment of viral PAMPs from RLRs, a process that is predicted to attenuate host cell innate immune activation. On the basis of previous data and that presented here, we propose that structural features of the MW, including components of the nuclear transport machinery, establish a selective permeability barrier between the surrounding cytosol and viral replication and assembly centers within the MW [8, 23, 37, 38, 57]. We show here that this barrier facilitates viral infection by inhibiting the access of RLRs to regions within the MW. These conclusions are supported by our findings that the addition of NLS sequences to either RIG-I or MDA5, which allows these proteins to interface with the nuclear transport machinery, overcome the barrier that restricts their access to compartments within the MW. A consequence of NLS-mediated movement of RLRs into the MW is increased immune activation and the inhibition of viral replication.

Although there are significant differences in the organization and architecture of the membrane rearrangements induced by positive-strand RNA viruses, the general functions of these compartments are predicted to be conserved. Like HCV, we observed that RIG-I is also inhibited from accessing replication factories produced by HAV, which induces a perinuclear tubular-vesicular membrane replication complex. Similarly, previous studies have shown that viral replication factories produced in Tick-borne encephalitis virus-infected cells (invaginated vesicle or spherule-type) inhibit immune activation by concealing viral dsRNA from cytosolic RLRs [36]. Collectively, these observations support the conclusions that, despite their apparent structural variability, membrane rearrangements induced by different positive-strand RNA viruses are likely to perform a universal function of concealing PAMPs from host RLRs as a means of immune evasion. Moreover, the presence of nuclear pore complex components within membrane structures induced by HAV and Dengue virus lead us to conclude that the nuclear transport machinery may be commonly employed to regulate the movement of molecules into and out of compartments formed by these membrane structures [37].

### Physical segregation of viral replication and assembly in the MW

How replication/assembly sites in positive-strand RNA virus-infected cells establish, compartmentalize, and segregate various biochemical activities remains largely ill defined. Within the complex architecture of the MW, it has been suggested that positive-strand RNA viruses coordinate different viral processes, such as replication and assembly, by spatially segregating these processes in different compartments [48, 57, 58]. Current theories on HCV assembly and egress propose that HCV RNA moves with the core protein from replication compartments to sites of virus assembly, suggesting a spatial separation between these processes [64, 65]. This idea is supported by several previous studies which indicate that viral maturation and assembly is mediated by the trafficking of proteins, including the NS2/p7 complex and the NS3/4A complex, from replication compartments to lipid droplets, which initiates assembly of viral particles [52, 66–71]. However, visualization and characterization of these different compartments has been difficult. In this study, we used subcellular fractionation techniques to isolate membrane fractions that were enriched for either viral replication or assembly components (Fig 4). The predominance of the viral polymerase NS5B in microsome-containing fractions implies an enrichment of replication complexes, while the concentration of the core protein in a membrane fraction that has similar sedimentation and buoyant density characteristics to MAMs suggests that this membrane fraction is enriched for assembly complexes. Consistent with these observations, increased levels of viral RNA co-fractionate with the microsomal fraction while increased levels of infectious virus are associated with the MAM membrane fraction (Fig 4B). Further analysis of the subcellular fractions also revealed that host proteins which function in replication (PI4K) were found in the microsomal fraction and those involved in assembly (ApoE) were present in the MAM fraction (Fig 4C). These results support the existence of distinct membrane compartments contributing to the bulk of replication and assembly and show that subcellular fractionation can be used to separate and characterize these different viral compartments.

### Exclusion of RLRs from the HCV-induced MW

In addition to its role in establishing distinct compartments for viral replication and assembly within its boundaries, the MW has also been suggested to function more broadly as physical barrier between these processes and the surrounding cytoplasm. This physical separation has been proposed to suppress the innate immune response, at least in part, by limiting access of RLRs to viral RNA (reviewed in [24, 72]). In this study, we provide evidence that cytoplasmic RIG-I and MDA5 are restricted from entering regions of the cytoplasm occupied by various HCV proteins that serve as molecular markers for the MW (Fig 1 and S1B Fig). Moreover, probes specific for positive- or negative-strand viral RNA reveal that RIG-I is excluded from regions of the MW containing both replication and assembly complexes (Figs 1 and 3 and S1A Fig). These results provided direct experimental evidence that the MW can function to limit access of innate immune receptors to viral RNA.

An important corollary that arises from our detection of RLR exclusion from the MW is that the localization of RLRs in HCV-infected cells provides a useful exclusion marker for the MW. Ours and numerous previous studies using either electron microscopy or immunofluorescence microscopy have outlined the complexity of the MW and demonstrated the difficulties in defining the MW using individual viral or host protein markers (Fig 1)[8, 21, 23, 73, 74]. Based on the results presented in this study, we suggest that regions of functional compartmentalization within the MW can be more accurately defined by both the presence of specific HCV proteins or viral RNA, and the exclusion of RLRs (defined by ectopically expressed RIG-I-K270A), bulk ribosomes, and tubulin (Figs 1, 3 and 5). We predict that defining these regions of the MW can provide an important tool for the analysis host factors and their role in the viral life cycle.

Using the exclusion of RIG-I from cytoplasmic regions containing HCV proteins as a marker for the MW, we observed that there are numerous smaller regions within the cytoplasm that harbour HCV replication and assembly complexes. These results are consistent with recent EM-tomography and correlative light-EM studies that reveal the MW is not confined to a single region in the cytoplasm, but rather it is made up of multiple regions that have higher abundance of NS5A and are enriched for single membrane vesicles and DMVs in conjunction with ER and lipid droplets [8]. This report describes ~3–10 μm regions of the cytoplasm as MW, consistent with our observations that ectopically expressed RLRs are excluded from multiple cytoplasmic regions that generally ranged in size from ~0.5–10 μm in size (Figs 1 and 3). The nature of the barrier that defines the perimeter of multiple MW compartments and contributes to the exclusion of macromolecules such as PRRs is unclear. However, as discussed below, the nuclear transport machinery appears to facilitate access to these MW compartments.

We also used RIG-I exclusion and viral protein/RNA markers to examine the cellular location of viral protein production. A spatial separation of viral RNA replication and translation has been proposed based on evidence that various positive-strand RNA virus replication complexes are composed of ribosome-free membranes and observations that these processes interfere with one another [6, 11–13, 48, 57, 58]. Here, we show that bulk ribosomes have a similar subcellular localization to that of RIG-I in HCV-infected cells, supporting the view that a physical separation exists between translation and genome replication/assembly (Fig 5 and S3 Fig). Consistent with this, a portion of the viral positive-strand RNA is found in regions containing RIG-I and ribosomes (Fig 3B). These observations led us to conclude that viral RNA destined for translation may exit the MW. Such a mechanism would require that some fraction of newly synthesized viral RNA be exported from the MW for translation, while another pool is retained in the MW and targeted to viral assembly centers. As discussed below, we envisage that components of the nuclear transport machinery may contribute to these targeting events.

### Transport from the surrounding cytosol to viral replication sites

In addition to restricting access of RLRs, the MW is also predicted to function as a barrier to molecules required for virus replication including entry into the MW of newly synthesized viral proteins and the exiting of positive-strand RNA for translation. This segregation necessitates a transport mechanism capable of regulating traffic between compartments within the MW and the surrounding cytoplasm. We envision that a contributing factor to this barrier may be surrounding ER membranes, and we have previously proposed that NPCs positioned within these membranes could regulate transport of macromolecules into regions of the MW containing viral replication and assembly machinery [22, 37, 38]. Consistent with this idea, functional NPCs are detected in the ER (historically termed annulate lamellae) and we have reported that HCV-infected cells exhibit increased levels of NPC proteins in regions of the cytoplasm occupied by the MW [37, 75, 76]. We suggested that MW-associated NPCs would allow NLS-containing proteins, including several HCV proteins and nuclear factors important for viral infection, to move into and out of the MW. Here we have provided further evidence supporting a transport function for the MW-associated NPCs and soluble nuclear transport factors by demonstrating that RLRs, which are normally inhibited from accessing replication/assembly complexes, colocalize with HCV proteins when they are tagged with an NLS sequence (Fig 6). Additionally, we show that the colocalization between NLS-tagged RIG-I and HCV proteins can be inhibited by the addition of nuclear transport inhibitors (Fig 7), further suggesting a role for the nuclear transport machinery in allowing access to the MW. Importantly, we also show that the addition of NLS sequences to active RLRs stimulates immune responses in HCV-infected cells and has an inhibitory affect on viral replication (Fig 8F). These results are consistent with the NLS-RIG-I fusion protein gaining increased access to the MW and viral RNA. Thus, virus-induced recruitment of NPCs appears to contribute to selective transport into the MW and to the HCV immune evasion strategy.

### RIG-I immune activation in HCV infection

The previously described activation of RIG-I-mediated immune responses in HCV-infected cells indicates that at least a portion of the viral RNA is present in regions of the cytoplasm containing RIG-I (reviewed in [77]). We envisage several scenarios that might allow RIG-I to interact with viral RNA despite their largely segregated localization patterns in infected cells. First, previous analysis of the HCV-induced immune response showed that RIG-I-mediated signalling is activated at early time points after infection [29]. Thus, RIG-I activation in HCV-infected cells could occur through an interaction with viral RNA prior to the establishment of a fully compartmentalized MW. Alternatively, viral ssRNA, which can associate with and activate RIG-I in the absence of dsRNA, could passively or actively exit the MW to the surrounding cytosol, potentially for the purpose of translation, where it could be recognized by RIG-I and initiate immune activation [78]. In both models, immune evasion through concealment of PAMPs would not be sufficient to block all immune activation, which is likely why the virus employs a number of other immune evasion strategies that have been previously described (reviewed in [79, 80]). Interestingly, these models predict that RLRs could only access viral positive-strand RNA, while double-stranded viral RNA is protected in the MW. This may explain why it has been difficult to observe MDA5-mediated immune activation, as MDA5 does not recognize ssRNA [29, 78].

### Conclusions

Collectively, our observations provide new insights into the selective barrier established by the MW in HCV-infected cells. The exclusion of RLRs from regions of the MW supports a role for this structure in the suppression of the innate immune response. Moreover, our results further implicate components of the nuclear transport machinery in regulating movement of molecules across the MW barrier. This work also underscores the importance of further studies to understand the role of nuclear transport machinery in both the biogenesis and function of replication compartments cells infected with HCV and other positive-strand RNA viruses.

## Materials and Methods

### Cell culture, viral infection and transport inhibitor drugs

HEK293T, A549, Huh7.5, and Huh7 cells were maintained in DMEM (Sigma) containing 10% FBS (Sigma). For HCV infection, Huh7.5 cells were seeded at a density of 2.5 × 10^5^ cells/well in 6-well tissue culture plates, and 24 hours after plating, they were infected with 3 RNA genome equivalents of a serially passaged JFH-1 strain of HCV. For HAV infection, Huh7 cells were grown to 70% confluence and infected with HAV/p16 virus at an MOI of 0.1. Cells were analyzed by immunofluorescence 3 weeks after infection. Huh7 cells containing the JFH-1 strain subgenomic replicon (encoding NS3 through to the C-terminus of the HCV polyprotein; received from the lab of Ralf Bartenschlager) were maintained in DMEM containing 10% FBS and 500 mg/mL G418. For the expression of constructs in HCV infected cells, cells were infected followed by transfection (as described below) at the indicated time after infection. For analysis of nuclear transport inhibition, uninfected and HCV-infected cells harbouring RIG-I expression constructs were treated with 40 μM importazole or 25 μM ivermectin for 3 hours.

### Quantitative real-time PCR (qPCR)

For analysis of intracellular RNA transcript levels, total RNA was extracted from cells using Trizol (Invitrogen, 15596018) and cDNA was synthesized using random primers (Invitrogen, 48190–011) and superscript II (Invitrogen, 18064014) according to the manufacturers specifications. Primers for qPCR were designed using Primer3 software and primer sequences are supplied in S1 Table. PCR efficiency for each primer was determined using the slope of a standard curve derived from qPCR analysis of cDNA serial dilutions. qPCR was done using a SYBR green super mix (Quanta, 95070–500) on a Stratagene Mx3005p real time PCR machine. To obtain the relative abundance of specific RNAs from each sample, cycle threshold (ct) values were corrected for the specific PCR efficiency of the primer, and normalized to hypoxanthine phosphoribosyltransferase 1 (HPRT) transcript levels. For analysis of HCV RNA levels in subcellular fractions, RNA was isolated from 200 μl of the media obtained from infected cells using a High Pure Viral Nucleic Acid Kit (Roche, 11858874001). The cDNA was synthesized using superscript III (Invitrogen, 18080044) and an HCV specific primer (see HCV reverse primer sequence S1 Table). qPCR was done using TaqMan Master Mix with HCV primers and labeled probe (S1 Table).

### Expression constructs and transfection

Expression constructs for production of FLAG-tagged RIG-I (pEF-Tak-RIG-I and pEF-Tak-RIG-I(K270A)) were prepared by first preparing PCR products of the entire RIG-I open reading frame from pEF-BOS RIG-I and pEF-BOS RIG-I (K270A) [78] into pEF-Tak encoding tandem FLAG epitope sequences in frame with the RIG-I protein under control of the elongation factor 1 promoter. RIG-I K270 was first prepared by site-directed mutagenesis using the Q5 mutagenesis kit (New England Biolabs) of the WT RIG-I sequence. PCR and mutagenic primers, and cloning methods are available upon request. Expression constructs for the production of GFP-tagged RIG-I (pEGFP-RIG-I, pEGFP-RIG-I(K270A), pEGFP-NLS-RIG-I, and pEGFP-NLS-RIG-I(K270A)) were produced by PCR amplification of RIG-I from the pEF-Tak-RIG-I constructs followed by cloning into the pEGFP-C1 vector. The NLS sequence used was the well-characterized NLS derived from the SV40 large T antigen. Expression constructs for the production of MDA5 were produced by first reverse transcribing MDA5 mRNA from U937 cell extracts to form cDNA followed by PCR amplification using specific primers using the One-Step RT-PCR System (Invitrogen, 10928–042). Amplified MDA5 DNA was then cloned into a pcDNA3.1/nV5-DEST expression vector (Invitrogen, 12290010) using the gateway cloning system (Invitrogen). The MDA5(I923V) mutation was introduced by site-directed mutagenesis using the QuickChange Lightning mutagenesis kit (Agilent Technologies). The primes used for PCR amplification and site-directed mutagenesis are listed in S1 Table. Constructs were sequenced to confirm the proper mutation. Constructs were transfected into Huh7.5 cells using lipofectamine 2000 reagent (Invitrogen, 11668019) and expressed for 48 hours.

### Immunofluorescence and western blotting

Immunofluorescence and western blotting were done as previously described [81]. Further details and a list of primary and secondary antibodies are provided in the extended Materials and Methods (S1 Text). Pearson’s correlation coefficients were used to determine the total overlap between two fluorescent signals. These numbers were calculated as previously described using the Coloc2 plugin for ImageJ [82]. Percent overlap between positive- or negative-strand probes and either RIG-I or ribosomes was determined using Manders colocalization coefficients. For graphs shown in the figures and percentages presented in the text, all imaging analysis was done on at least 10 different images with at least 15 individual cells. Quantification of the nuclear and cytoplasmic IRF-3 fluorescence levels in Fig 8 was done using ImageJ

### Subcellular fractionation

Subcellular fractionation was performed as previously described [37, 83, 84]. For details, see extended Materials and Methods (S1 Text).

### Specific infectivity and infectious titer assays

Subcellular fractions were serially diluted and added to Huh7.5 cells grown in optical 96 well plates. 2 days after infection, viral focus-forming units were determined by indirect immunofluorescence microscopy using antibodies specific to HCV core protein. The values for specific infectivity were calculated by dividing the number of Focus forming units by the total number of HCV RNA copies added to the cells (FFU/HCV RNA copy). The values for infectious titer represent the number of focus forming units per ml in the culture medium harvested from each of the coinfected samples. The values for specific infectivity and infectious titer show an average over a count of 6 wells, and each experiment was repeated 3 times.

### Accession numbers

The NCBI accession numbers of the proteins and genes described in the paper are: RIG-I- NM_014314.3, MDA5- NM_022168.3, Calnexin- NM_001746.3, VDAC1- NM_003374.2, S6- NM_001010.2, Nup98- NM_016320, lamin B- NM_032737, ICAM- NM_000201.2, CXCL10- NM_001565.3, IRF-1- NM_002198.2, βTubulin—NM_006000, ApoE—NM_001302688.1, PI4K—NM_058004.3, HPRT—NM_000194.2, HCV JFH—HM049503, and HCV H77—JX472013.

## Supporting Information

S1 Fig Characterization of RLR localization compared to membranous web markers. A) Huh7 cells or Huh7 cells harbouring the JFH-1 subgenomic replicon were transfected with constructs encoding V5-tagged MDA5 and incubated for 2 days. The localization of NS5A (red) and V5-tagged MDA5 (green) was evaluated by indirect immunofluorescence microscopy using antibodies specific for NS5A and the V5 epitope tag. Pearson’s correlation coefficients shown in the merge images were calculated using Coloc2 software in ImageJ and represent overlap between the red and green fluorescent channels in the indicated image. B) HCV-infected Huh7.5 cells were transfected with a construct encoding for FLAG-tagged Rig-I-K207A 2 days after HCV infection. On day 4 after infection, cells were fixed and incubated with antibodies directed against the FLAG epitope (grey) and HCV core (green). DNA was detected with DAPI (blue). Samples were visualized using a DeltaVision OMX Imaging System. Pearson’s correlation coefficients shown in the merge images were calculated using Coloc2 software in ImageJ and represent overlap between the grey and green fluorescent channels in the indicated image. C) Uninfected or HCV-infected Huh7.5 cells were transfected with a construct encoding for FLAG-tagged Rig-I-K207A 2 days after HCV infection. On day 4 after infection, cells were incubated with BODIPY (green) followed by incubation with antibodies directed against the FLAG epitope (grey) and HCV core (red). In both panels DNA was detected with DAPI (blue) and scale bars represent 5 μm. Boxed regions in the middle row of both panels outline the area of magnification presented in the bottom rows. All images were obtained using a confocal microscope.
(TIF)

S2 Fig Localization of viral proteins and viral RNA in HCV-infected cells. Uninfected or HCV-infected Huh7.5 cells were transfected with constructs encoding FLAG-tagged Rig-I-K207A 2 days after HCV infection. On day 4 after infection, cells were probed with antibodies directed against the FLAG epitope (grey) and either HCV core (panel A, green) or NS5A (panel B, green). DNA probes (Affymetrix) complementary to either positive-strand (panel A, red) or negative-strand HCV RNA (panel B, red) were then hybridized to the samples using the manufacturers protocol. DNA was stained with DAPI (blue) and scale bars represent 5 μm. Boxed regions in the middle row of both panels outline the area of magnification presented in the bottom rows. All images were obtained using a confocal microscope.
(TIF)

S3 Fig Exclusion of ribosomes from viral replication compartments. Uninfected or HCV-infected Huh7.5 cells were transfected with a construct encoding FLAG-tagged RIG-I-K270A 2 days after HCV infection. On day 4 after infection, cells were probed with antibodies directed against the FLAG-tagged RIG-I-K270A (red or green as indicated) and antibodies directed against either HCV core (red) or the S6 ribosomal protein (green). DNA was stained with DAPI (blue) and scale bars represent 5 μm. All images were obtained using a confocal microscope. Pearson’s correlation coefficients shown in the merge images were calculated using Coloc2 software in ImageJ and represent overlap between the red and green fluorescent channels in the indicated image.
(TIF)

S4 Fig Localization of NLS-GFP reporter to the membranous web. Uninfected or HCV-infected Huh7.5 cells were transfected 2 days after infection with a construct encoding a chimeric protein consisting of an N-terminal SV-40 NLS sequence followed by two tandemly-repeated GFP molecules. On day 4 after infection, the NLS-GFP reporter was visualized by fluorescence microscopy (green) and its location compared to tubulin (grey) and HCV Core (red) detected by immunofluorescence microscopy. DNA was detected with DAPI (blue) and scale bars represent 5 μm. Boxed regions in the middle row of both panels outline the area of magnification presented in the bottom rows. All images were obtained using a confocal microscope.
(TIF)

S5 Fig Construct expression levels and quantification of immune transcript levels following expression of RIG-I containing constructs. Uninfected or HCV-infected Huh7.5 cells were transfected with constructs encoding for RIG-I-GFP, NLS-RIG-I-GFP, SLN-RIG-I-GFP, or NLS-RIG-I-K270A-GFP 1 day after HCV infection. Three days after infection, cells were harvested using TRIzol reagent and RNA transcript levels were determined. A) Transcript levels for each of the RIG-I constructs in HCV-infected cells was determined using qPCR using primers specific to the GFP tag. B) Transcript levels for each of the indicated immune gene transcripts in uninfected Huh7.5 cells were determined by qPCR using specific primers. For all panels, the values presented are relative to HPRT transcript levels in Huh7.5 cells.
(TIF)

S1 Text Extended Materials and Methods. (DOCX)

S1 Table Real time qPCR primers used in this study. (DOC)
