# Supplementary figures and images for: The Hepatitis C Virus-Induced Membranous Web and Associated Nuclear Transport Machinery Limit Access of Pattern Recognition Receptors to Viral Replication Sites
Source: PLoS Pathog. 2016 Feb 10;12(2):e1005428. doi: 10.1371/journal.ppat.1005428 (PMC4749181; doi:10.1371/journal.ppat.1005428)

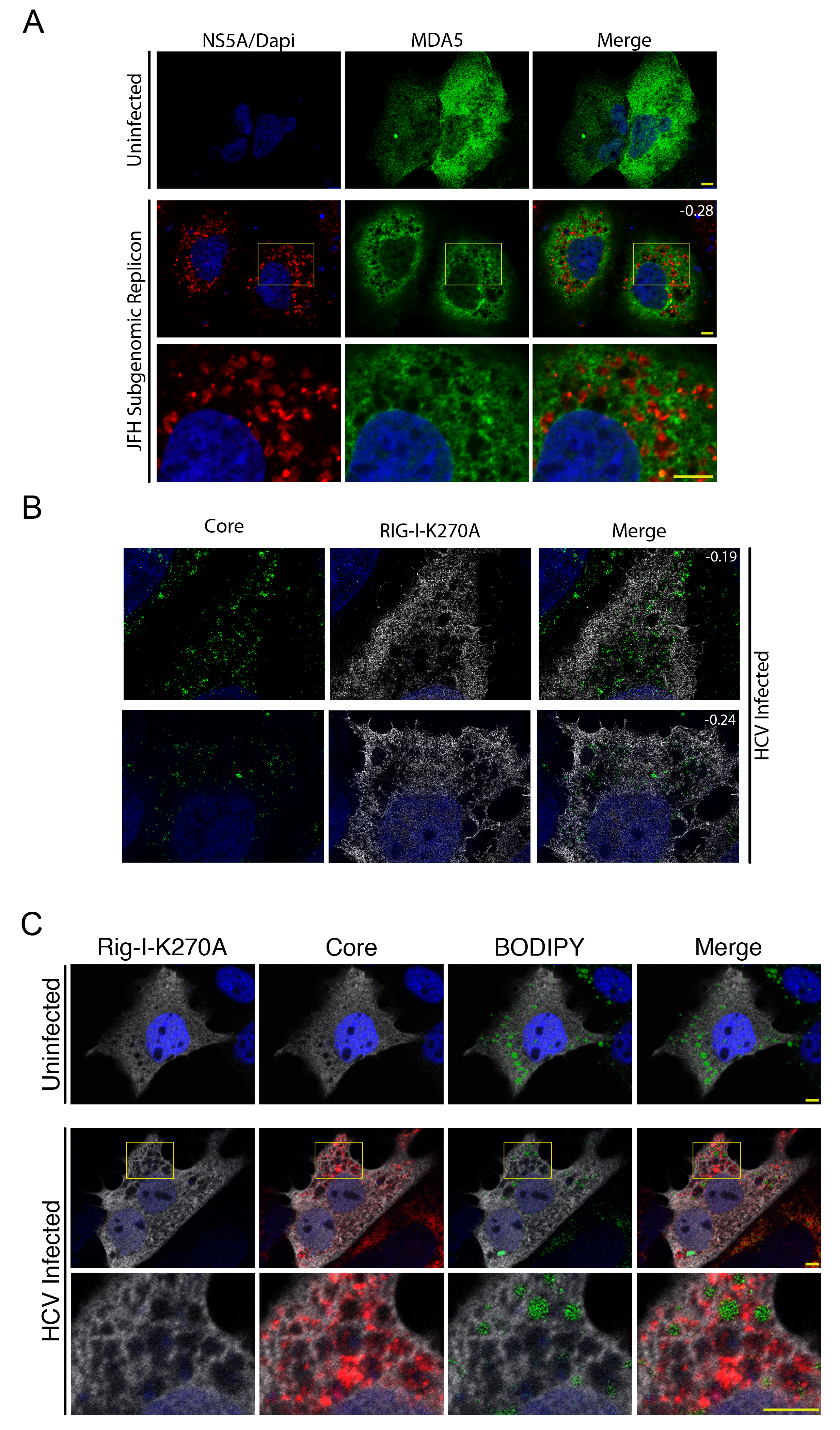

Supplement: S1 Fig — A) Huh7 cells or Huh7 cells harbouring the JFH-1 subgenomic replicon were transfected with constructs encoding V5-tagged MDA5 and incubated for 2 days. The localization of NS5A (red) and V5-tagged MDA5 (green) was evaluated by indirect immunofluorescence microscopy using antibodies specific for NS5A and the V5 epitope tag. Pearson’s correlation coefficients shown in the merge images were calculated using Coloc2 software in ImageJ and represent overlap between the red and green fluorescent channels in the indicated image. B) HCV-infected Huh7.5 cells were transfected with a construct encoding for FLAG-tagged Rig-I-K207A 2 days after HCV infection. On day 4 after infection, cells were fixed and incubated with antibodies directed against the FLAG epitope (grey) and HCV core (green). DNA was detected with DAPI (blue). Samples were visualized using a DeltaVision OMX Imaging System. Pearson’s correlation coefficients shown in the merge images were calculated using Coloc2 software in ImageJ and represent overlap between the grey and green fluorescent channels in the indicated image. C) Uninfected or HCV-infected Huh7.5 cells were transfected with a construct encoding for FLAG-tagged Rig-I-K207A 2 days after HCV infection. On day 4 after infection, cells were incubated with BODIPY (green) followed by incubation with antibodies directed against the FLAG epitope (grey) and HCV core (red). In both panels DNA was detected with DAPI (blue) and scale bars represent 5 μm. Boxed regions in the middle row of both panels outline the area of magnification presented in the bottom rows. All images were obtained using a confocal microscope. (TIF) [file ppat.1005428.s001.tif]

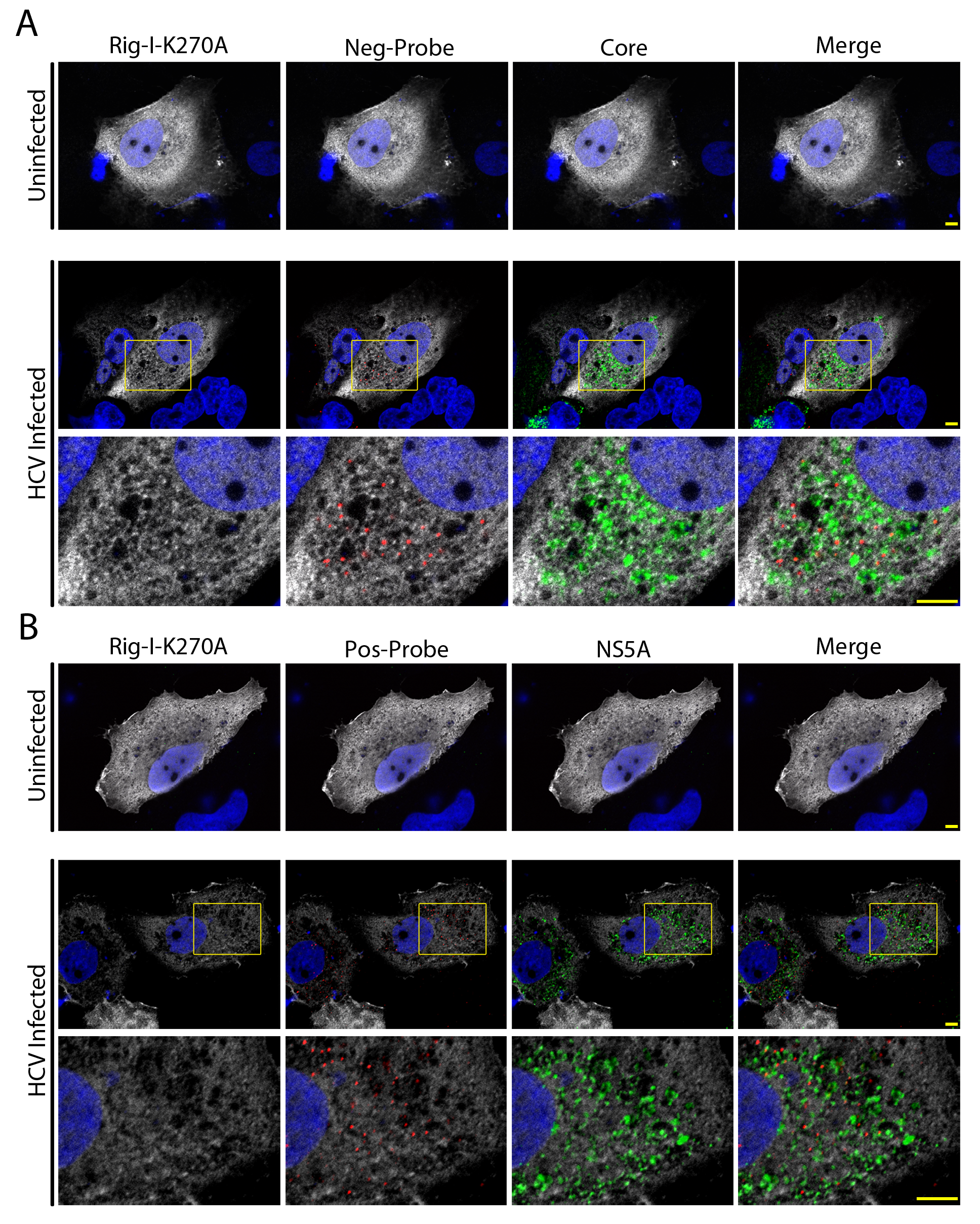

Supplement: S2 Fig — Uninfected or HCV-infected Huh7.5 cells were transfected with constructs encoding FLAG-tagged Rig-I-K207A 2 days after HCV infection. On day 4 after infection, cells were probed with antibodies directed against the FLAG epitope (grey) and either HCV core (panel A, green) or NS5A (panel B, green). DNA probes (Affymetrix) complementary to either positive-strand (panel A, red) or negative-strand HCV RNA (panel B, red) were then hybridized to the samples using the manufacturers protocol. DNA was stained with DAPI (blue) and scale bars represent 5 μm. Boxed regions in the middle row of both panels outline the area of magnification presented in the bottom rows. All images were obtained using a confocal microscope. (TIF) [file ppat.1005428.s002.tif]

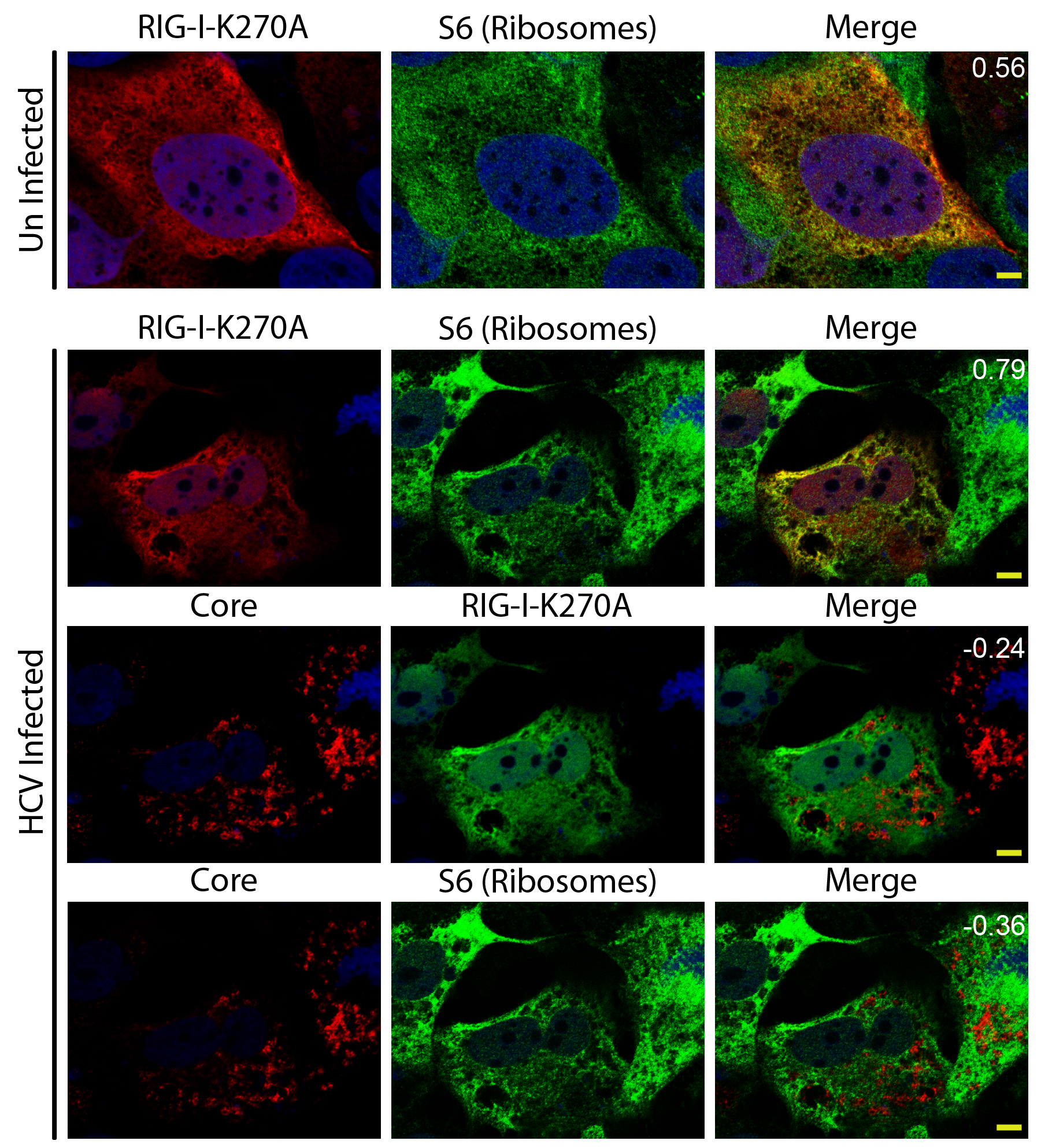

Supplement: S3 Fig — Uninfected or HCV-infected Huh7.5 cells were transfected with a construct encoding FLAG-tagged RIG-I-K270A 2 days after HCV infection. On day 4 after infection, cells were probed with antibodies directed against the FLAG-tagged RIG-I-K270A (red or green as indicated) and antibodies directed against either HCV core (red) or the S6 ribosomal protein (green). DNA was stained with DAPI (blue) and scale bars represent 5 μm. All images were obtained using a confocal microscope. Pearson’s correlation coefficients shown in the merge images were calculated using Coloc2 software in ImageJ and represent overlap between the red and green fluorescent channels in the indicated image. (TIF) [file ppat.1005428.s003.tif]

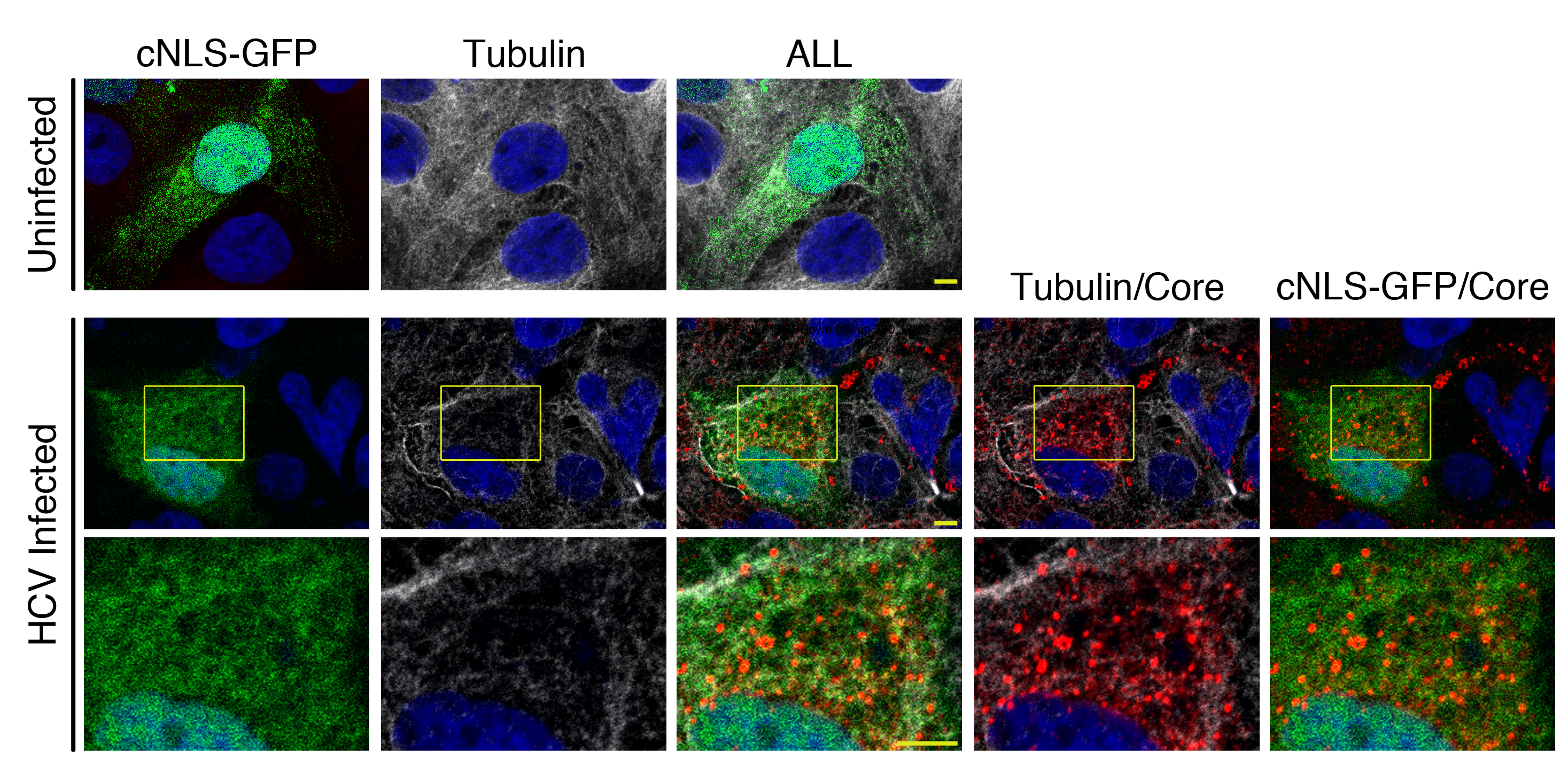

Supplement: S4 Fig — Uninfected or HCV-infected Huh7.5 cells were transfected 2 days after infection with a construct encoding a chimeric protein consisting of an N-terminal SV-40 NLS sequence followed by two tandemly-repeated GFP molecules. On day 4 after infection, the NLS-GFP reporter was visualized by fluorescence microscopy (green) and its location compared to tubulin (grey) and HCV Core (red) detected by immunofluorescence microscopy. DNA was detected with DAPI (blue) and scale bars represent 5 μm. Boxed regions in the middle row of both panels outline the area of magnification presented in the bottom rows. All images were obtained using a confocal microscope. (TIF) [file ppat.1005428.s004.tif]

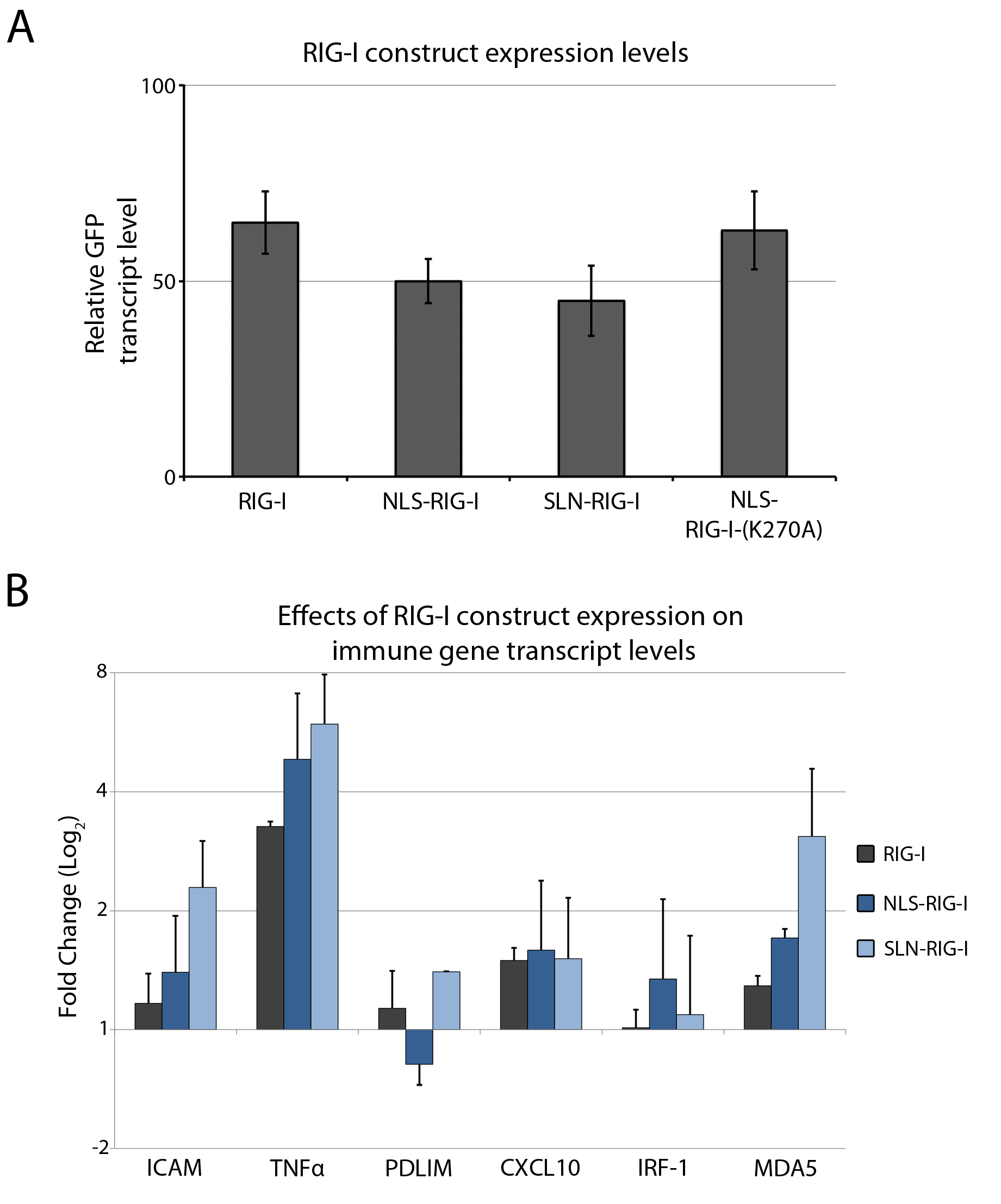

Supplement: S5 Fig — Uninfected or HCV-infected Huh7.5 cells were transfected with constructs encoding for RIG-I-GFP, NLS-RIG-I-GFP, SLN-RIG-I-GFP, or NLS-RIG-I-K270A-GFP 1 day after HCV infection. Three days after infection, cells were harvested using TRIzol reagent and RNA transcript levels were determined. A) Transcript levels for each of the RIG-I constructs in HCV-infected cells was determined using qPCR using primers specific to the GFP tag. B) Transcript levels for each of the indicated immune gene transcripts in uninfected Huh7.5 cells were determined by qPCR using specific primers. For all panels, the values presented are relative to HPRT transcript levels in Huh7.5 cells. (TIF) [file ppat.1005428.s005.tif]
