# Supplementary material for: The Hepatitis C Virus-Induced Membranous Web and Associated Nuclear Transport Machinery Limit Access of Pattern Recognition Receptors to Viral Replication Sites
Source: PLoS Pathog. 2016 Feb 10;12(2):e1005428. doi: 10.1371/journal.ppat.1005428 (PMC4749181; doi:10.1371/journal.ppat.1005428)
Supplement: S1 Text — (DOCX) [file ppat.1005428.s006.docx]

**EXTENDED MATERIALS AND METHODS**

**Antibodies**

Antibodies directed against Nup98 [[1](#_ENREF_1)], Lamin B [[2](#_ENREF_2)], HAV capsid [[3](#_ENREF_3)], HCV NS4B [[4](#_ENREF_4)], IRF-3 [[5](#_ENREF_5)], HCV NS5A [[6](#_ENREF_6)] and HCV NS5B [[7](#_ENREF_7)] have been previously described. Commercially available antibodies were used to detect α-tubulin (Sigma-Aldrich, T6074), VDAC1 (Abcam, ab15895), the FLAG epitope (Abcam, ab1257), S6 ribosomal subunit (Signalway Antibody Co. Ltd., 21225-100), calnexin (Enzo, ADI-SPA-860) dsRNA (Scicons, J2), HCV core (Thermo Scientific, MA1-080), HCV core (Abcam, ab58713), HCV NS3 (Millipore, MAB8691), ApoE (Millipore, AB947), PI4K (Cell Signaling Technology, #4902), and the V5 epitope (Abcam, ab27671). For western blotting, the horseradish peroxidase (HRP)-conjugated and fluorescent probe conjugated secondary antibodies used to detect primary antibodies included: donkey anti–rabbit IgG-HRP (GE Healthcare, NA934V), sheep anti–mouse IgG-HRP (GE Healthcare, NA931V), Alexa Fluor 750 goat anti-rabbit IgG (Invitrogen, A21039), and Alexa Fluor 680 goat anti-mouse IgG (Invitrogen, A21057). For indirect immunofluorescence microscopy, primary antibodies were detected using Alexa Fluor 488 donkey anti-rabbit IgG (Life Technologies, A21206), Alexa Fluor 488 donkey anti-mouse IgG (Life Technologies, A21202), Alexa Fluor 594 donkey anti-mouse IgG (Life Technologies, A21203), Alexa Fluor 594 donkey anti-rabbit IgG (Life Technologies, A11012), Alexa Fluor 594 donkey anti-goat IgG (Life Technologies, A11058) Alexa Fluor 647 donkey anti-mouse IgG (Life Technologies, A-31571), Alexa Fluor 647 donkey anti-rabbit IgG (Life Technologies, A-31573) and Alexa Fluor 647 Goat anti-mouse IgG (Life Technologies, A21236) secondary antibodies.

**Western Blotting**

Cells were lysed using SDS-PAGE sample buffer, followed by sonication and denaturation at 95°C for 10 minutes. 10 µg of total protein from each sample was resolved by SDS-PAGE and transferred to nitrocellulose. Membranes were blocked with PBS-T (PBS - pH 7.4 containing 0.1% Tween-20) containing 5% skim milk for 2 hours at room temperature, followed by incubation with anti-sera containing primary antibodies overnight at 4°C. Following incubation with primary antibodies, membranes were washed and incubated for 2 hours with either the HRP-conjugated or fluorescent probe-conjugated secondary antibodies described above. For HRP-conjugated secondary antibodies, membranes were washed in PBS-T followed by initiation of HRP chemiluminescence with ECL detection reagent (GE Healthcare, RPN2106) and the signal was detected using Fuji RX film (Fujifilm, 47410 08399). For fluorescent labeled blots, membranes were sequentially washed in PBS-T, PBS, and water. Secondary antibody binding was detected with an Odyssey infrared imaging system (Licor). Quantification of protein levels detected with the Licor system was done using Odyssey V3.0 software.

**Immunofluorescence**

Huh7.5 cells grown on glass cover slips were fixed with 3.2% formaldehyde (Sigma, F8775-500ML) at room temperature for 10 minutes then permeabilized in 0.2% Triton X-100 (VWR, CA97062-208) for 2 minutes at room temperature. To visualize lipid droplets, samples were incubated with BODIPY 493/503 at 1 μg/ml (Invitrogen, D3922) for 15 minutes at room temperature after fixation. Cover slips were then blocked in 2.5% skim milk for 2 hours and incubated with the indicated primary antibodies at 4°C overnight. Samples were then washed and incubated with secondary antibodies for 45 minutes at room temperature, followed by mounting onto microscope slides using Dapi-Fluoromount-G mounting media (Southern BioTech, 0100-20).

For *in situ* hybridization of RNA probes, the QuantiGene® ViewRNA ISH Cell Assay (Affymetrix) was used as per the manufactures protocol. Briefly, following incubation with secondary antibodies as described above, cells were re-fixed for 30 minutes in 3.2% formaldehyde at room temperature. Samples were then incubated with detergent solution provided by Affymetrix for 5 minutes at room temperature. Probe sets (from Affymetrix) specific for either the positive- or negative-strand of the HCV genome were the hybridized to the samples by incubating at 40°C for 3 hours. The primary probe signal was amplified by sequential hybridization of pre-amplifier and amplifier DNA probes each at 40°C for 30 minutes. Samples were then incubated with fluorescent labeled probes, specific to the amplifier probes, for 30 minutes at 40°C. Coverslips were mounted onto microscope slides using Dapi-Fluoromount-G (Southern BioTech, 0100-20) and stored in the dark at 4°C for up to 5 days.

Epifluorescence images were obtained using an Axio Observer Z1 microscope (Carl Zeiss, Inc.) using a 63x/1.40 NA Oil UPlanS-Apochromat objective lens (Carl Zeiss Inc.). Images taken on the epifluorescent microscope were deconvolved using Axiovision software (Carl Zeiss, Inc.). Confocal images were obtained with a LSM 710 Axio Observer microscope (Carl Zeiss Inc.) using a 63x/1.40 NA Oil DIC Plan-Apochromat objective. Images were acquired as a z-stack series (with a distance of 0.24μm between slices) and are represented as a z-projection. Super resolution images were taken using a DeltaVision OMX microscope with a 60X/1.42 Oil lens and structured illumination (GE Healthcare Life Science). Structured illumination image processing was done using SoftWoRx software (GE Healthcare Life Science). Z-projection and merging of channels was done using ImageJ software (National Institutes of Health). Pearson’s colocalization coefficients were calculated as previously described using the Coloc2 plugin for ImageJ [[8](#_ENREF_8)]. Photoshop 5.0 (Adobe) software was used to adjust brightness and contrast levels for individual images and assemble images into figures.

**Subcellular fractionation**

Huh7.5 cells were infected with HCV as described above. On day 4 after infection, cells were removed from plates with trypsin and lysed in isolation buffer (IB; 225 mM Manitol, 75 mM Sucrose, 0.1 mM EGTA, 30 mM Tris-HCL - pH 7.4) using a Balch homogenizer at 4°C. The remaining procedures were preformed on ice or at 4°C. The nuclear fraction was isolated from the lysate by low speed centrifugation at 600 x g for 10 minutes. Crude mitochondria and associated membranes were pelleted from the supernatant by centrifugation at 7 000 x g for 10 minutes followed by resuspension in 10 ml IB and centrifugation at 7 000 x g for 10 minutes . The supernatant was collected and the membranes in microsomal fraction were pelleted from the supernatant by centrifugation at 150 000 x g. for 60 minutes. The microsomal membrane pellet was resuspended in mitochondrial resuspension buffer (MRB)(250mM Mannitol, 5mM HEPES - pH 7.4). The mitochondria and associated membranes pellet described above was resuspended in MRB and layered onto 10 mls of Percoll Isolation Medium (PIM) (225 mM Mannitol, 25 mM HEPES - pH 7.4, 1 mM EGTA, 30% v/v Percoll). Samples were then centrifuged at 95 000 x g for 30 minutes to separate the MAM fraction from the mitochondrial fraction. The MAM fraction was isolated from the gradient and centrifuged for 10 minutes at 6 300 x g and the resulting supernatant (containing the MAM) was diluted to 10 mls with MRB. These samples were then centrifuged for 60 minutes at 100 000 x g to pellet the MAM fraction. The pellet containing the MAM fraction was resuspended in MRB. The mitochondria containing fraction (isolated from the Percoll gradient) was further purified by centrifugation for 10 minutes at 6 300 x g. The pellet was resuspended in IB and centrifuged again at 6 300 x g for 10 minutes to pellet mitochondria. The enriched mitochondrial pellet was resuspended in MRB. The total amount of protein in each sample was calculated using a DC™ protein assay kit (BioRad, 500-0116). SDS-PAGE sample buffer was added to each fraction and protein contents were evaluated by SDS-PAGE and western blotting.

**SUPPLEMENTAL REFERENCES**

1. Mitchell JM, Mansfeld J, Capitanio J, Kutay U, Wozniak RW. Pom121 links two essential subcomplexes of the nuclear pore complex core to the membrane. J Cell Biol. 2010;191(3):505-21. Epub 2010/10/27. doi: 10.1083/jcb.201007098. PubMed PMID: 20974814; PubMed Central PMCID: PMC3003318.

2. Chaudhary N, Courvalin JC. Stepwise reassembly of the nuclear envelope at the end of mitosis. J Cell Biol. 1993;122(2):295-306. Epub 1993/07/01. PubMed PMID: 8391536; PubMed Central PMCID: PMC2119651.

3. Feng Z, Hensley L, McKnight KL, Hu F, Madden V, Ping L, et al. A pathogenic picornavirus acquires an envelope by hijacking cellular membranes. Nature. 2013;496(7445):367-71. Epub 2013/04/02. doi: 10.1038/nature12029. PubMed PMID: 23542590; PubMed Central PMCID: PMC3631468.

4. Paul D, Hoppe S, Saher G, Krijnse-Locker J, Bartenschlager R. Morphological and biochemical characterization of the membranous hepatitis C virus replication compartment. J Virol. 2013;87(19):10612-27. Epub 2013/07/26. doi: 10.1128/JVI.01370-13. PubMed PMID: 23885072; PubMed Central PMCID: PMC3807400.

5. Sumpter R, Jr., Loo YM, Foy E, Li K, Yoneyama M, Fujita T, et al. Regulating intracellular antiviral defense and permissiveness to hepatitis C virus RNA replication through a cellular RNA helicase, RIG-I. J Virol. 2005;79(5):2689-99. PubMed PMID: 15708988.

6. Lindenbach BD, Evans MJ, Syder AJ, Wolk B, Tellinghuisen TL, Liu CC, et al. Complete replication of hepatitis C virus in cell culture. Science. 2005;309(5734):623-6. PubMed PMID: 15947137.

7. Wilson JA, Jayasena S, Khvorova A, Sabatinos S, Rodrigue-Gervais IG, Arya S, et al. RNA interference blocks gene expression and RNA synthesis from hepatitis C replicons propagated in human liver cells. Proc Natl Acad Sci. 2003;100(5):2783-8. Epub 2003/02/21. doi: 10.1073/pnas.252758799. PubMed PMID: 12594341; PubMed Central PMCID: PMC151418.

8. Manders EM, Stap J, Brakenhoff GJ, van Driel R, Aten JA. Dynamics of three-dimensional replication patterns during the S-phase, analysed by double labelling of DNA and confocal microscopy. Journal of cell science. 1992;103 ( Pt 3):857-62. Epub 1992/11/01. PubMed PMID: 1478975.
