# Supplementary material for: The Hepatitis C Virus-Induced Membranous Web and Associated Nuclear Transport Machinery Limit Access of Pattern Recognition Receptors to Viral Replication Sites
Source: PLoS Pathog. 2016 Feb 10;12(2):e1005428. doi: 10.1371/journal.ppat.1005428 (PMC4749181; doi:10.1371/journal.ppat.1005428)
Supplement: S1 Table — (DOC) [file ppat.1005428.s007.doc]

**Table S1. Real time qPCR primers used in this study**

|  | **Primer Name** | **Sequence** |
| --- | --- | --- |
| 1 | IRF-1 Forward | 5'-ggattccagccctgatacct-3' |
| 2 | IRF-1 Reverse | 5'-cctgcatgtagcctggaact-3' |
| 3 | ICAM Forward | 5'-accagagccaggagacactg-3' |
| 4 | ICAM Reverse | 5'-acctcggtcccttctgagac-3' |
| 5 | TNFα Forward | 5'-aatggcgtggagctgaga-3' |
| 6 | TNFα Reverse | 5'-agccttggcccttgaaga-3' |
| 7 | PDLIM Forward | 5'-ctacacctgtgccgactgtg-3' |
| 8 | PDLIM Reverse | 5'-ctccaaagcttccagtgacaa-3' |
| 9 | CXCL10 Forward | 5'-actgtacgctgtacctgcatca-3' |
| 10 | CXCL10 Reverse | 5'-atgatctcaacacgtggacaaa-3' |
| 11 | MDA5 Forward | 5'-gagctggacacagcagtgagt-3' |
| 12 | MDA5 Reverse | 5'-tgccactgtggtagcgataa-3' |
| 13 | HPRT Forward | 5'-cctggcgtcgtgattagtg-3' |
| 14 | HPRT Reverse | 5'-acaccctttccaaatcctcag-3' |
| 15 | HCV Forward | 5'-tctgcggaaccggtgagta-3' |
| 16 | HCV Reverse | 5'-gtgtttcttttggtttttctttgaggtttagg-3' |
| 17 | HCV probe | 5'-FAM-cacggtctacgagacctcccggggcac-TAMARA-3' |
